# Profiling and Quantifying Differential Gene Transcription Provide Insights into Ganoderic Acid Biosynthesis in *Ganoderma lucidum* in Response to Methyl Jasmonate

**DOI:** 10.1371/journal.pone.0065027

**Published:** 2013-06-07

**Authors:** Ang Ren, Meng-Jiao Li, Liang Shi, Da-Shuai Mu, Ai-Liang Jiang, Qin Han, Ming-Wen Zhao

**Affiliations:** Microbiology Department, College of Life Sciences, Nanjing Agricultural University, Key Laboratory of Microbiological Engineering of Agricultural Environment, Ministry of Agriculture, Nanjing, Jiangsu, P.R. China; Soonchunhyang University, Korea, Republic Of

## Abstract

*Ganoderma lucidum* is a mushroom with traditional medicinal properties that has been widely used in China and other countries in Eastern Asia. Ganoderic acids (GA) produced by *G. lucidum* exhibit important pharmacological activities. Previous studies have demonstrated that methyl jasmonate (MeJA) is a potent inducer of GA biosynthesis and the expression of genes involved in the GA biosynthesis pathway in *G. lucidum*. To further explore the mechanism of GA biosynthesis, cDNA-Amplified Fragment Length Polymorphism (cDNA-AFLP) was used to identify genes that are differentially expressed in response to MeJA. Using 64 primer combinations, over 3910 transcriptionally derived fragments (TDFs) were obtained. Reliable sequence data were obtained for 390 of 458 selected TDFs. Ninety of these TDFs were annotated with known functions through BLASTX searching the GenBank database, and 12 annotated TDFs were assigned into secondary metabolic pathways by searching the KEGGPATHWAY database. Twenty-five TDFs were selected for qRT-PCR analysis to confirm the expression patterns observed with cDNA-AFLP. The qRT-PCR results were consistent with the altered patterns of gene expression revealed by the cDNA-AFLP technique. Additionally, the transcript levels of 10 genes were measured at the mycelium, primordia, and fruiting body developmental stages of *G. lucidum*. The greatest expression levels were reached during primordia for all of the genes except cytochrome b2 reached its highest expression level in the mycelium stage. This study not only identifies new candidate genes involved in the regulation of GA biosynthesis but also provides further insight into MeJA-induced gene expression and secondary metabolic response in *G. lucidum*.

## Introduction

Medicinal mushrooms are viewed as a rich source of therapeutically useful biologically active agents. There are approximately 700 species of higher basidiomycetes that have been found to possess significant pharmacological activities [1]. For several thousand years, *Ganoderma lucidum* (Ling-Zhi in Chinese and Reishi in Japanese) has been widely used in Asia as a home remedy to treat minor disorders and promote vitality and longevity [2]. Numerous studies have revealed that the primary active ingredients of *G. lucidum* are polysaccharides and the secondary metabolites ganoderic acids (GAs) [3], [4]. Most GAs have important medicinal value, such as the regulation of osteoclast genesis [5], the inhibition of cholesterol synthesis [3] and tumor growth [6], and protection of the liver [7]. However, despite the important pharmacological potential of GAs, low GA yield from both field cultivation and fermentation limits its wide-spread use.

Many attempts have been made to increase GA biosynthesis. Those works can be divided into two branches. Most reports focus on the environmental conditions during fermentation. The optimal medium (carbon source, nitrogen source, mineral source, and initial pH) was elucidated by an orthogonal design study that tested one factor at a time [8]. By studying the effect of the fed-batch fermentation process (pH-shift and dissolved oxygen tension-shift) on the GA content, strategies were identified that resulted in a significant synergistic enhancement of GA accumulation [9]. Recently, the use of an inducer to enhance the activity components in fungi fermentation has drawn great interest [10], [11]. For GA production, methyl jasmonate, phenobarbital and H_2_O_2_ were added to culture medium to increase the GA content [12]–[14]. However, due to the unclear mechanism of ganoderic acid biosynthesis, determining the optimal fermentation conditions and screening an effective inducer to produce maximum quantities of GA are still a trial-and-error process.

Isotopic tracer experiments have demonstrated that GA, a type of terpenoid, is synthesized via the mevalonate pathway [15], [16]. The genes that encode the proteins involved in the GA biosynthesis pathway have been cloned and characterized, and the regulation of the expression levels of these genes has been investigated under different environmental conditions to determine the relationship between GA biosynthesis and the expression of these genes [17]–[20]. Recent studies have demonstrated that the over-expression of these biosynthetic genes results in an enhanced accumulation of GA in *G. lucidum* [21]–[23]. Although these reports indicated that increased GA biosynthesis may result from the up-regulation of GA biosynthesis genes, how specific environmental conditions induce GA biosynthesis through the GA biosynthetic pathway remains unclear. Therefore, research on the GA biosynthesis mechanism has concentrated on the mevalonate pathway.

MeJA is a ubiquitous small signaling molecule in the plant kingdom. Environmental stresses, such as wounding or pathogen attack, can trigger MeJA production [24], [25]. In plants, MeJA induces stomatal closure, monoterpenoid indole alkaloids and isoprenoid biosynthesis, and defense response pathogens by activating reactive oxygen species, the MAPK signal pathway, or the calcium-dependent protein kinase signal pathway [25]–[28]. In fungi, MeJA is involved in the modulation of *Cryptococcus laurentii* and *Penicillium expansum* growth [29] and the regulation of Aflatoxin B1 biosynthesis by *Aspergillus parasiticus* [30], [31]. The same phenomenon is observed in *G. lucidum*; when different concentrations of MeJA were added to the culture, the GA contents were improved [12]. Moreover, the transcript levels of the genes *hmgr*, *fps*, and *sqs* in the GA biosynthesis pathway were up-regulated in response to MeJA. However, the signaling pathways initiated by MeJA to regulate GA biosynthesis and gene expression remain unknown.

In this study, differentially expressed transcripts were screened in the MeJA-treated mycelium using cDNA-AFLP to gain insights into the regulatory mechanisms of GA biosynthesis in response to MeJA. The differentially expressed transcripts were sequenced and classified, and their expression patterns were analyzed. For some of the regulated genes, quantitative real-time polymerase chain reaction (qRT-PCR) was used to confirm the expression patterns observed with cDNA-AFLP. In addition, the transcript levels of some of the candidate genes were investigated at various developmental stages of *G. lucidum*.

## Results

### Isolation of Differentially Expressed Genes

**Figure 1:**
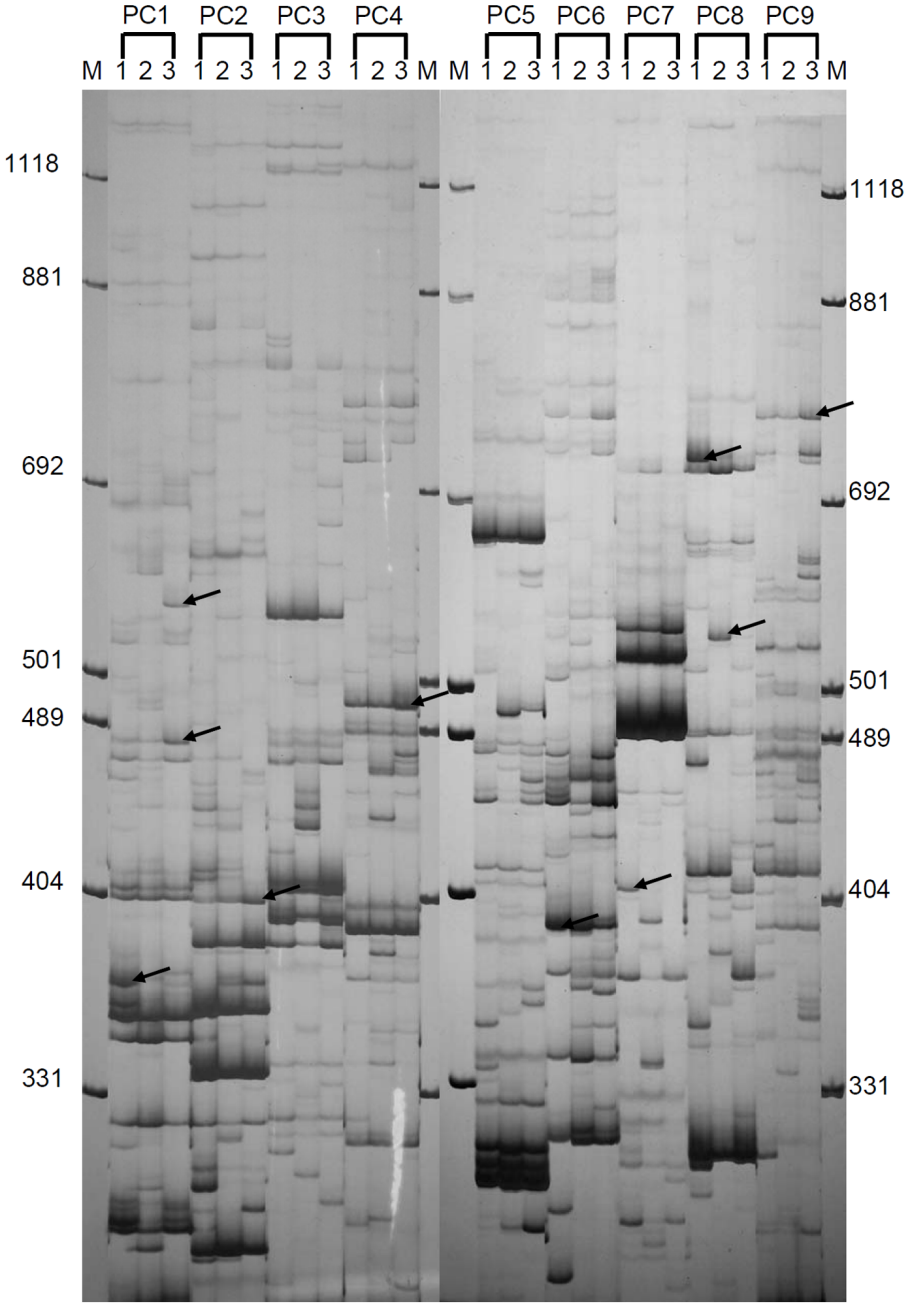
cDNA-AFLP analysis of transcripts in response to MeJA treatment in *G. lucidum*. cDNA-AFLP silver-stained polyacrylamide gels with 9 primer combinations (PC) amplifying differentially expressed genes in *G. lucidum* treated with 0, 50 and 200 µM MeJA, respectively. The combinations of primers used are indicated according to the codes reported in Table S4. The molecular weight marker sizes are indicated on both sides. Arrows indicate some of the differentially expressed transcript-derived fragments.

To select a suitable restriction enzyme combination for cDNA-AFLP analysis of *G. lucidum*, several enzyme combinations were tested; the combination of *Eco*R I and *Mse* I produced an acceptable range of fragment sizes (Figure 1).

A total of 64 primer combinations was used to selectively amplify the expressed genes. Differentially expressed transcript-derived fragments were extracted from the gel and used as templates for re-amplification by PCR. The cDNA-AFLP fragments were highly reproducible, as evidenced by the similar band intensities observed in the three biological replications. All of the visible TDFs between 150 and 800 bp were counted. Of the total 3910 transcript-derived fragments obtained using cDNA-AFLP with 64 primer pairs, 919 (23.5%) displayed altered expression patterns after MeJA induction; 703 were up-regulated, and 216 were down-regulated. Reliable sequences were obtained for 390 TDFs out of 458 TDFs selected for further analysis. Sequence data from this article have been deposited in GenBank, Accession Numbers: JZ163375- JZ163764. According to the genomic sequence of *G. lucidum* [32], [33], the distributions of 390 TDFs were analyzed as shown in Figure S1. Because our knowledge of gene functions in *G. lucidum* is relatively limited, only 90 of the sequenced genes were associated with known functions, as determined by BLAST searching the GenBank database (Table 1 and Table 2). The sites of known functional TDFs on chromosomes were analyzed as shown in Table 2 and Figure S2. Several differentially expressed genes showed homology to genes encoding transcription factors and genes involved in metabolism, gene regulation, signal transduction, stress defense, protein trafficking and protein degradation (Table 2).

**Table 1:** Classification of TDFs from the cDNA-AFLP result in functional categories.

Function	TDFs, %	U, %	D, %
Metabolism/energy	36 (9.23)	26 (6.67)	10 (2.56)
Transcription	14 (3.59)	7 (1.79)	7 (1.79)
Protein synthesis/fate	15 (3.85)	12 (3.08)	3 (0.77)
Signal transduction	10 (2.56)	5 (1.28)	5 (1.28)
Transport facilitation	11 (2.82)	6 (1.54)	5 (1.28)
Defense/cell organization	4 (1.03)	4 (1.03)	0 (0.00)
Unclassified proteins	151 (38.72)	105 (26.92)	46 (11.79)
No hits	149 (38.21)	81 (20.77)	68 (17.44)
Total	390 (100.0)	248 (63.59)	142 (36.41)

The number and frequency (in parentheses) of 390 TDFs from the cDNA-AFLP result with defined functional categories annotated is indicated. U, up-regulation; D, down-regulation.

**Table 2:** Transcript derived fragments (TDFs) from *G. lucidum* with homologies to other known protein.

No.	TDF	Size	Homologue^a^	Max Score^a^	Max ident^a^	E value^a^	Expression^b^	Chromosomes Site^c^
**Metabolism/energy**								
1	TDF006	271	glucosidase I (Coprinopsis cinerea)	140	71%	6.00E-32	U	No hits found
2	TDF015	468	NAD-dependent deacetylase (Puccinia graminis )	120	42%	9.00E-26	D	Chr6
3	TDF070	466	ceramidase (Coprinopsis cinerea)	243	77%	9.00E-63	U	Chr13
4	TDF080	365	1,3-beta-glucan synthase (Laccaria bicolor)	171	80%	3.00E-41	D	Chr11
5	TDF096	275	aryl-alcohol oxidase (Coprinopsis cinerea)	95.9	70%	2.00E-18	U	No hits found
6	TDF300	375	phosphoglycerate kinase (Coprinopsis cinerea)	365	79%	2.00E-123	D	No hits found
7	TDF138	677	anthranilate synthase (Coprinopsis scobicola)	310	68%	3.00E-98	U	Chr5
8	TDF142	494	saccharopine dehydrogenase (Coprinopsis cinerea)	194	79%	6.00E-59	D	Chr3
9	TDF143	461	2-methylcitrate dehydratase (Coprinopsis cinerea)	192	74%	7.00E-57	U	Chr5
10	TDF047	612	cytochrome b2 (Coprinopsis cinerea)	180	66%	1.00E-43	U	Chr3
11	TDF099	262	acetolactate synthase (Neosartorya fischeri)	86.3	55%	1.00E-15	U	Chr7
12	TDF375	194	calcium transporting ATPase (Coprinopsis cinerea)	63.2	100%	6.00E-15	D	Chr13
13	TDF153	478	aspartate ammonia lyase (Coprinopsis cinerea)	272	84%	3.00E-88	U	Chr1
14	TDF376	538	lipase/esterase (Coprinopsis cinerea)	155	43%	1.00E-40	U	Chr1
15	TDF113	600	acetyl-CoA acetyltransferase (Coprinopsis cinerea)	302	74%	2.00E-80	U	Chr1
16	TDF115	252	biotin-[acetyl-CoA-carboxylase] ligase (Laccaria bicolor)	113	70%	9.00E-24	D	Chr11
17	TDF160	718	cytochrome P450 (Postia placenta)	239	55%	7.00E-74	U	Chr2
18	TDF161	511	glycoside hydrolase family 31 protein (Serpula lacrymans)	225	62%	3.00E-67	U	Chr3
19	TDF381	309	flavin-containing monooxygenase (Aspergillus niger)	104	49%	3.00E-24	U	Chr12
20	TDF313	287	cytochrome-b5 reductase (Coprinopsis cinerea)	99.4	78%	6.00E-24	U	GaLu96scf_50
21	TDF314	327	Hexokinase (Coprinopsis cinerea)	151	81%	3.00E-41	U	Chr11
22	TDF019	265	cytochrome b5 (Phanerochaete chrysosporium)	123	76%	2.00E-34	U	Chr1
23	TDF353	278	heparinase II/III family protein (Coprinopsis cinerea)	128	56%	6.00E-32	D	Chr3
24	TDF355	219	cytochrome P450 (Postia placenta)	147	65%	5.00E-40	U	Chr1
25	TDF243	238	pyruvate carboxylase (Laccaria bicolor)	142	84%	1.00E-38	U	Chr6
26	TDF195	478	fumarase (Scheffersomyces stipitis)	269	82%	2.00E-87	U	Chr3
27	TDF322	268	Cytochrome P450 like TBP (Medicago truncatula)	74.3	65%	4.00E-17	U	Chr10
28	TDF323	252	pyruvate kinase (Coprinopsis cinerea)	151	85%	7.00E-42	U	Chr3
29	TDF325	414	malate dehydrogenase (Coprinopsis cinerea)	197	78%	1.00E-60	D	Chr1
30	TDF364	552	cytochrome P450 (Dichomitus squalens)	248	66%	8.00E-77	U	Chr12
31	TDF367	288	sulfate permease (Laccaria bicolor)	144	71%	3.00E-38	D	Chr5
32	TDF307	239	syntaxin-like protein (Laccaria bicolor)	117	73%	2.00E-29	U	Chr4
33	TDF256	260	ERG27-3-keto sterol reductase (Piriformospora indica)	50.8	42%	3.00E-06	U	Chr2
34	TDF291	143	formaldehyde dehydrogenase (Taiwanofungus camphoratus)	83.2	89%	2.00E-18	D	Chr1
35	TDF223	170	glycoside hydrolase family 3 protein (Serpula lacrymans)	75.1	61%	2.00E-15	U	Chr4
36	TDF338	209	ATP synthase subunit gamma (Coprinopsis cinerea)	85.1	90%	2.00E-18	U	Chr11
**Transcription**								
37	TDF009	336	nucleotide binding protein, putative (Candida dubliniensis)	103	50%	6.00E-21	D	Chr3
38	TDF020	330	transcription factor (Stereum hirsutum)	211	90%	3.00E-62	U	Chr1
39	TDF042	271	pre-mRNA splicing factor prp1 (Coprinopsis cinerea)	113	63%	1.00E-23	D	Chr7
40	TDF058	551	IMP-specific 5′-nucleotidase 1 (Uncinocarpus reesii)	221	59%	5.00E-56	U	Chr3
41	TDF081	316	epsilon DNA polymerase (Coprinopsis cinerea)	156	71%	9.00E-37	D	Chr1
42	TDF318	302	eukaryotic translation initiation factor 6 (Postia placenta)	156	95%	1.00E-46	U	Chr8
43	TDF094	379	DNA-directed RNA polymerase II subunit (Coprinopsis cinerea)	206	91%	1.00E-51	U	Chr6
44	TDF114	466	translation initiation factor 3 subunit 3 (Coprinopsis cinerea)	171	71%	5.00E-41	U	Chr3
45	TDF050	398	Chromo domain protein MRG15 (Piriformospora indica)	174	47%	1.00E-50	D	Chr12
46	TDF049	374	translation elongation factor 1a (Schizophyllum commune)	266	94%	6.00E-85	U	Chr3
47	TDF370	233	rRNA intron-encoded homing endonuclease (Medicago truncatula)	137	49%	1.00E-34	D	Chr5
48	TDF341	303	RWD domain-containing protein (Laccaria bicolor)	141	57%	8.00E-40	D	No hits found
49	TDF156	340	argonaute-like protein (Laccaria bicolor)	140	66%	1.00E-36	D	Chr11
50	TDF390	320	transcription factor (Stereum hirsutum)	204	90%	8.00E-60	U	Chr1
**Protein synthesis/fate**								
51	TDF297	312	peptidylprolyl isomerase (Datisca glomerata)	186	82%	4.00E-59	U	Chr9
52	TDF136	444	ubiquitin-protein ligase (Coprinopsis cinerea)	208	68%	5.00E-61	U	Chr4
53	TDF299	304	histone H2B (Coprinopsis cinerea)	176	100%	2.00E-54	U	Chr2
54	TDF145	561	60S ribosomal protein L10 (Postia placenta)	333	95%	2.00E-115	U	Chr10
55	TDF303	439	profilin (Laccaria bicolor)	186	74%	1.00E-58	U	Chr9
56	TDF151	501	SNARE protein SED5	192	78%	2.00E-58	U	Chr3
57	TDF164	405	mitochondrial endopeptidase (Serpula lacrymans)	198	66%	2.00E-58	U	Chr7
58	TDF312	272	histone H4 (Coprinopsis cinerea)	160	100%	3.00E-50	U	Chr6
59	TDF383	181	signal peptidase 21 kDa subunit (Coprinopsis cinerea)	113	91%	4.00E-31	U	Chr11
60	TDF347	333	Ubiquitin (Camponotus floridanus)	246	98%	2.00E-82	U	Chr3
61	TDF385	286	mitochondrial 50S ribosomal protein L5 (Postia placenta)	84.7	84%	1.00E-18	D	Chr7
62	TDF321	429	40S ribosomal protein S11 (Postia placenta)	263	91%	2.00E-88	U	Chr3
63	TDF336	505	60S ribosomal protein L32 (Postia placenta)	248	98%	1.00E-83	U	Chr3
64	TDF340	240	p47 protein isoform c (Coprinopsis cinerea)	107	77%	3.00E-26	D	Chr12
65	TDF293	256	histone deacetylase RPD3 (Coprinopsis cinerea)	152	84%	1.00E-42	D	Chr12
**Signal transduction**								
66	TDF013	297	CMGC/MAPK/JNK protein kinase (Coprinopsis cinerea )	104	58%	4.00E-21	D	Chr4
67	TDF193	736	Rho2 GTP binding protein (Ustilago maydis)	127	88%	1.00E-50	D	Chr2
68	TDF040	299	cAMP-dependent protein kinase Akt (Coprinopsis cinerea)	90.5	49%	7.00E-17	D	Chr1
69	TDF051	669	histidine kinase (Schizophyllum commune)	116	62%	3.00E-24	U	Chr6
70	TDF052	547	protein kinase activator Mob2 (Coprinopsis cinerea)	203	78%	8.00E-51	U	Chr7
71	TDF304	299	serine/threonine kinase receptor associated protein (Coprinopsis cinerea)	196	84%	3.00E-60	U	No hits found
72	TDF158	580	CMGC/GSK protein kinase (Coprinopsis cinerea)	159	81%	6.00E-45	U	Chr4
73	TDF165	675	rho small monomeric GTPase (Coprinopsis cinerea)	268	74%	6.00E-89	D	Chr2
74	TDF008	848	Ras2 (Cryptococcus neoformans)	89	69%	8.00E-35	D	Chr8
75	TDF333	394	signal recognition particle binding protein (Coprinopsis cinerea)	187	90%	1.00E-53	U	No hits found
**Transport facilitation**								
76	TDF078	444	glycerol uptake facilitator (Talaromyces stipitatus)	105	45%	2.00E-21	D	Chr5
77	TDF083	441	copper chaperone TahA (Trametes versicolor)	117	83%	8.00E-25	U	Chr8
78	TDF159	548	vacuolar sorting protein (Ajellomyces capsulatus)	153	43%	2.00E-43	D	Chr3
79	TDF187	490	oligopeptide transporter (Laccaria bicolor)	266	76%	3.00E-83	U	Chr10
80	TDF342	350	inorganic phosphate transporter (Laccaria bicolor)	99	76%	2.00E-22	D	Chr8
81	TDF048	218	nuclear transport factor 2 (Coprinopsis cinerea)	174	80%	5.00E-54	D	Chr10
82	TDF360	315	vacuole protein (Cryptococcus neoformans)	96.3	64%	1.00E-23	U	Chr3
83	TDF327	167	COPII-coated vesicle protein (Coprinopsis cinerea)	85.9	85%	3.00E-20	U	Chr1
84	TDF384	259	NifU-like protein c (Coprinopsis cinerea)	126	74%	3.00E-34	D	Chr3
85	TDF316	294	t-complex protein 1 (Coprinopsis cinerea)	162	73%	8.00E-46	U	Chr1
86	TDF264	521	vacuolar membrane protein (Cryptococcus gattii)	97.8	73%	6.00E-28	U	Chr4
**Defense/cell organization**								
87	TDF122	674	cell division control protein 23 (Coprinopsis cinerea)	236	65%	2.00E-60	U	Chr5
88	TDF176	346	hsp70-like protein (Coprinopsis cinerea)	91.7	71%	4.00E-20	U	Chr10
89	TDF179	280	microtubule associated protein (Coprinopsis cinerea)	99	75%	4.00E-23	U	No hits found
90	TDF129	448	catalase (Trametes versicolor)	216	66%	1.00E-63	U	Chr3

a Based on highest BLASTX match with an E value lower than 1e-^04^.

b U, up-regulation; D, down-regulation.

c The distribution of TDFs on chromosomes in *G. lucidum* genome.

### Gene Sequence Analysis

The annotation approach was based on sequence similarity searches in the GenBank database. The 390 TDFs were subjected to a BLASTX search against the NCBI non-redundant protein database using the default parameters. The results revealed that 241 TDFs (61.8%) had significant sequence similarities to known proteins (eValue≤10^−5^): 90 TDFs (23.08%) had significant sequence similarity to classified proteins, 151 TDFs (38.72%) had sequence similarity to unclassified proteins; and the remaining 149 TDFs (38.21%) failed to match any proteins in the database. It was noted that the information about the genomes or transcriptomes of this species was needed in-depth analysis. Of the 90 TDFs, 45.6% were homologous to *Coprinopsis cinere*, 11.1% were homologous to *Laccaria bicolor* and 7.8% were homologous to *Postia placenta*.

**Figure 2:**
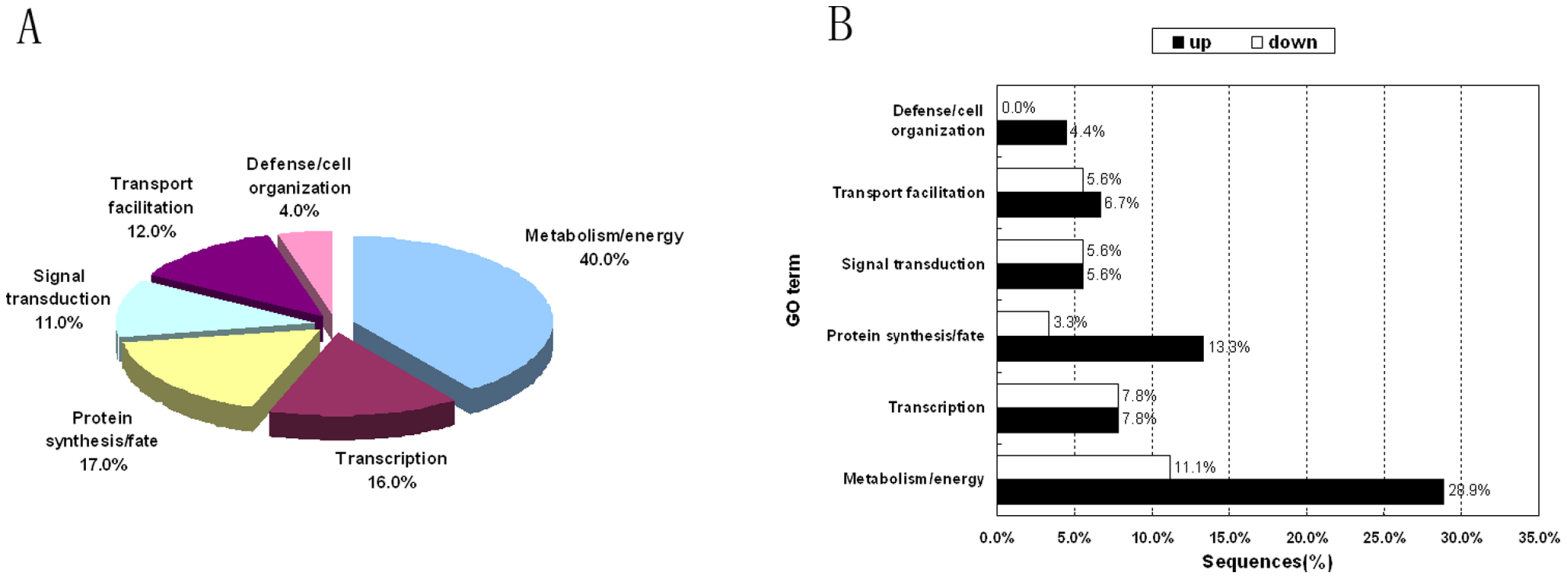
Percentages of 90 known functional TDFs with functional categories. A. The functional classification of transcript-derived fragments (90 TDFs) from *G. lucidum* in response to MeJA. The percentages of differentially expressed genes in the 90 known functional sequences assigned to various functional categories. B. Ninety TDFs in biological function categories showing differential expression patterns in *G*. *lucidum*. The percentages of different functional categories of up- and down-regulated sequences in the 90 known functional sequences were significantly different (P<0.05).

Gene ontology (GO) assignments describe gene products in terms of their associated molecular functions, biological processes and cellular components. Blast2GO (B2G) is a bioinformatic tool for GO-based DNA or protein sequence annotation [34]. The 90 TDFs were submitted to Blast2GO, and 75 were successfully annotated (Table S1). Figure 2A shows the percentages of differentially expressed genes in the 90 known functional sequences assigned to various functional categories. Of these, 40.0% of the annotations were related to ‘metabolism/energy’, 17.0% were related to ‘protein synthesis/fate’, and 16.0% were related to ‘transcription’.

Figure 2B shows that the percentages of different functional categories of up- and down-regulated sequences in the 90 known functional sequences were significantly different (P<0.05). In the metabolism/energy group and the protein synthesis/fate group, the percentages of up-regulated genes (28.9% and 13.3%, respectively) were increased compared with the percentages of down-regulated genes (11.1% and 3.3%, respectively). In the transcription term and the signal transduction term, the percentages of up-regulated genes (7.8% and 5.6%, respectively) were equal to the percentages of down-regulated genes (7.8% and 5.6%, respectively). Interestingly, all of the differentially expressed genes were up-regulated in the defense/cell organization group (4.4%).

KEGG provides a reference knowledge base for linking genomes to life through the process of PATHWAY mapping. In this study, the 90 annotated TDFs were blasted against the KEGG database. Thirty of these TDFs were highly homologous to some protein, and 21 were assigned to the metabolic pathways in the database (Table S2). Interestingly, 12 TDFs were assigned to secondary metabolic pathways, specifically, the biosynthesis of triterpenes, terpenoids and steroids.

### Quantitative RT-PCR Analysis of MeJA-induced Differentially Expressed TDFs in *G. lucidum*

**Figure 3:**
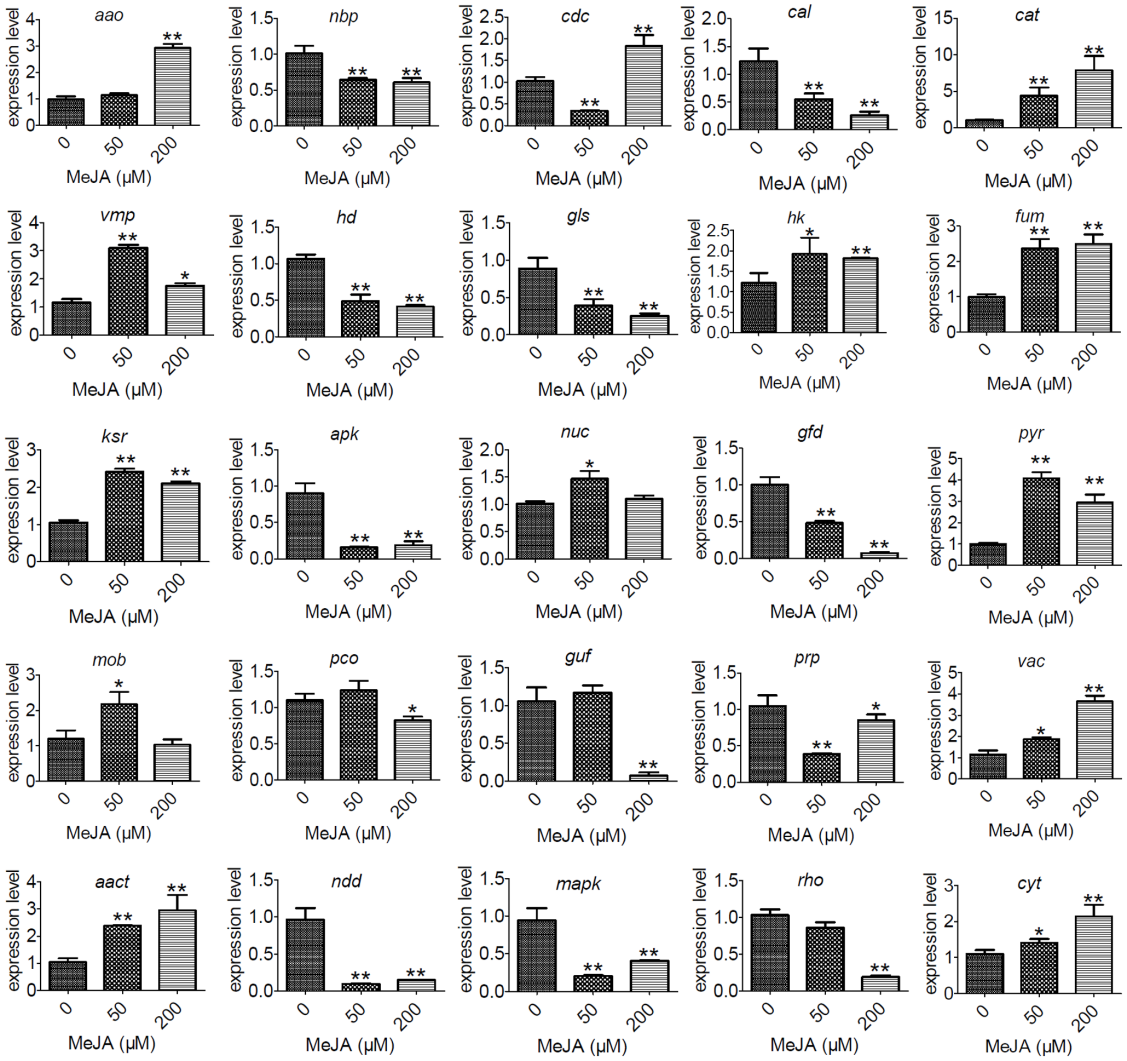
qRT-PCR analysis of 25 selected TDFs in *G. lucidum*. Expression of 25 selected genes treated with 0, 50 and 200 µM MeJA, respectively. aao (TDF096) aryl-alcohol oxidase, nbp (TDF009) nucleotide binding protein, cdc (TDF122) cell division control protein, cal (TDF375) calcium transporting ATPase, cat (TDF129) catalase, vmp (TDF264) vacuolar membrane protein, hd (TDF293) histone deacetylase, gls (TDF080) glucosidase I, hk (TDF051) histidine kinase, fum (TDF195) fumarase, ksr (TDF256) ERG27-3-keto sterol reductase, apk (TDF040) cAMP-dependent protein kinase, nuc (TDF058) IMP-specific 5′-nucleotidase 1, gfd (TDF291) glutathione-dependent formaldehyde dehydrogenase, pyr (TDF323) pyruvate kinase, mob (TDF052) protein kinase activator, pco (TDF243) pyruvate carboxylase, guf (TDF078) glycerol uptake facilitator, prp (TDF042) pre-mRNA splicing factor, vac (TDF360) vacuole protein, aact (TDF113) acetyl-CoA acetyltransferase, ndd (TDF015) NAD-dependent deacetylase, mapk (TDF013) CMGC/MAPK/JNK protein kinase, rho (TDF165) small monomeric GTPase, cyt (TDF047) cytochrome b2. All samples were examined in triplicate. For all genes represented in this figure, the P value was <0.05 or 0.01 (*p<0.05 and **p<0.01).

Co-expression analysis, which is based on the premise that a set of genes involved in a biological process is co-expressed under given conditions, has been successfully used to identify novel genes involved in secondary metabolism [35]. To verify the correlation between the expression of differentially expressed TDFs and MeJA induction, quantitative RT-PCR analysis was performed for 25 TDFs, which involved in metabolism (glucosidase I (gls), glutathione-dependent formaldehyde dehydrogenase (gfd), fumarase (fum), NAD-dependent deacetylase (ndd), pyruvate carboxylase (pco), pyruvate kinase (pyr), ERG27-3-keto sterol reductase (ksr), aryl-alcohol oxidase (aao), catalase (cat), cytochrome b2 (cyt) and acetyl-CoA acetyltransferase (aact)), gene regulation (nucleotide binding protein (nbp), histone deacetylase (hd), pre-mRNA splicing factor (prp) and IMP-specific 5′-nucleotidase 1 (nuc)), signal transduction (cAMP-dependent protein kinase (apk), CMGC/MAPK/JNK protein kinase (mapk), small monomeric GTPase (rho), histidine kinase (hk) and protein kinase activator (mob)), cell organization (cell division control protein (cdc)) and trafficking (vacuolar membrane protein (vmp), vacuole protein (vac), calcium transporting ATPase (cal) and glycerol uptake facilitator (guf)). In Figure 3, nbp (TDF009), cal (TDF375), hd (TDF293), gls (TDF080), apk (TDF040), gfd (TDF291), guf (TDF078), prp (TDF042), ndd (TDF015), mapk (TDF013), and rho (TDF165) were down-regulated in response to MeJA treatment, whereas others were up-regulated. For hk (TDF051), mob (TDF052), nuc (TDF058), pyr (TDF323), pco (TDF243), ksr (TDF256), and vmp (TDF264), the highest levels of transcripts were observed with 50 µM MeJA. For aao (TDF096), cdc (TDF122), cat (TDF129), fum (TDF195), vac (TDF360), cyt (TDF047), and aact (TDF113), the highest levels of transcripts were observed with 200 µM MeJA. The qRT-PCR results are consistent with the altered expression patterns observed for these 25 genes using cDNA-AFLP (Figure S3).

### Variations in Gene Expression at Developmental Stages of *G. lucidum*

**Figure 4:**
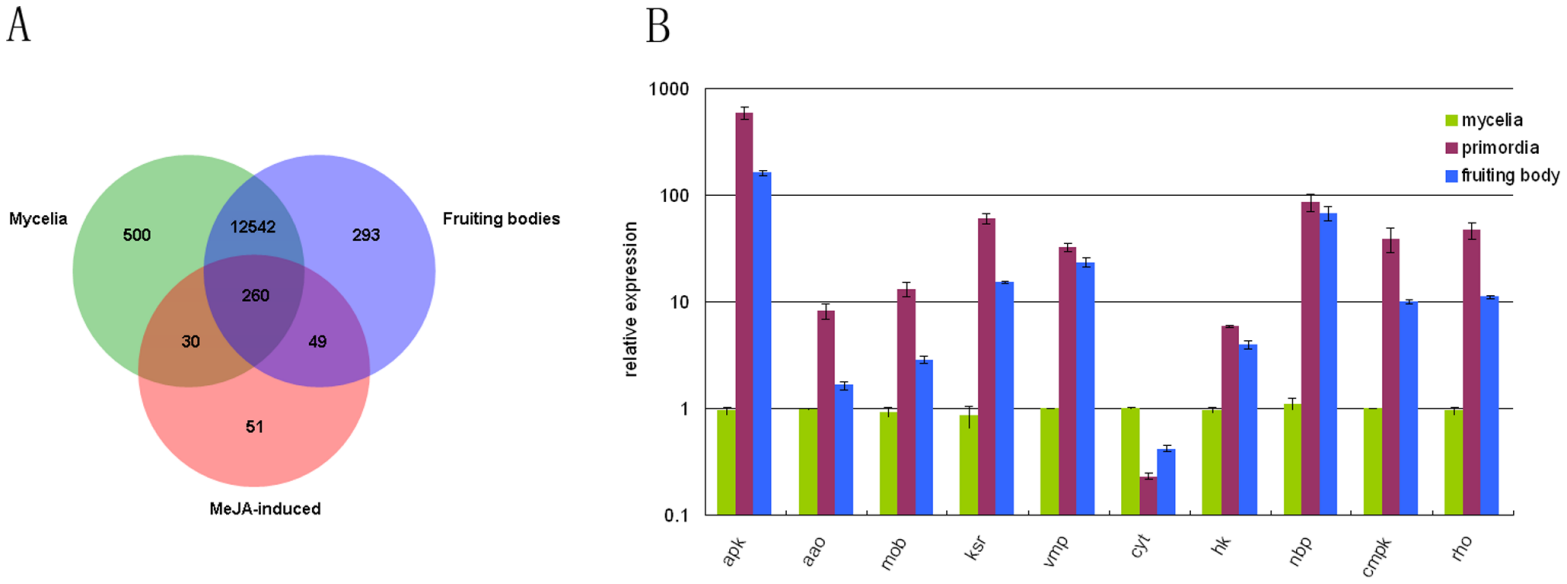
The expression of TDF genes in different development stages. A. Venn diagrams depicting the genes expressed across MeJA-induction and the different developmental stages. Data are derived from Table S3. B. The transcript levels of TDF genes under the developmental stages of *G. lucidum*. The X axis shows the abbreviation of genes. The full name of each gene is in the Figure 3 legends. All samples were examined in triplicate. For all genes represented in this figure, the P value was <0.01.

All of TDFs from MeJA-induced library were searched from mycelium or fruiting body EST library reported by Chen et al., 2012 [32] (Figure 4A and Table S3). In the three EST libraries, there are 260 genes accounted for the majority, including basic metabolism and signal transduction genes. There are 30 genes appeared in both mycelium library and MeJA-induced library, most of which are of unknown function genes. Forty-nine genes appear in both the fruiting EST library and MeJA-induced library, including ERG27-3-keto sterol reductase and cytochrome P450. Fifty genes only exist in MeJA-induced library, indicating that these genes inducing conditions expressed in methyl jasmonate, and may not be expressed or low expressed in mycelium and fruiting bodies, such as pre-mRNA splicing factor and catalase.

Varying amounts of GA are observed in different developmental stages in *G. lucidum.* A recent study reported that the GA level is highest during the primordium and fruiting body stages [32]. To further study the relationship between the differentially expressed genes and GA biosynthesis, the transcription levels of 10 genes were examined during the mycelium, primordium, and fruiting body developmental stages in *G. lucidum* (Figure 4B). Expression levels were the highest during primordium for TDF040 (apk, cAMP-dependent protein kinase), TDF096 (aao, aryl-alcohol oxidase), TDF052 (mob, protein kinase activator), TDF256 (ksr, ERG27-3-keto sterol reductase), TDF051 (hk, histidine kinase), TDF013 (mapk, CMGC/MAPK/JNK protein kinase), and TDF165 (rho, small monomeric GTPase). For TDF264 (vmp, vacuolar membrane protein) and TDF009 (nbp, nucleotide binding protein), expression levels were the highest during both the primordium and the fruiting body stages. Only TDF047 (cyt, cytochrome b2) showed a maximum expression level during the mycelium stage.

## Discussion

**Figure 5:**
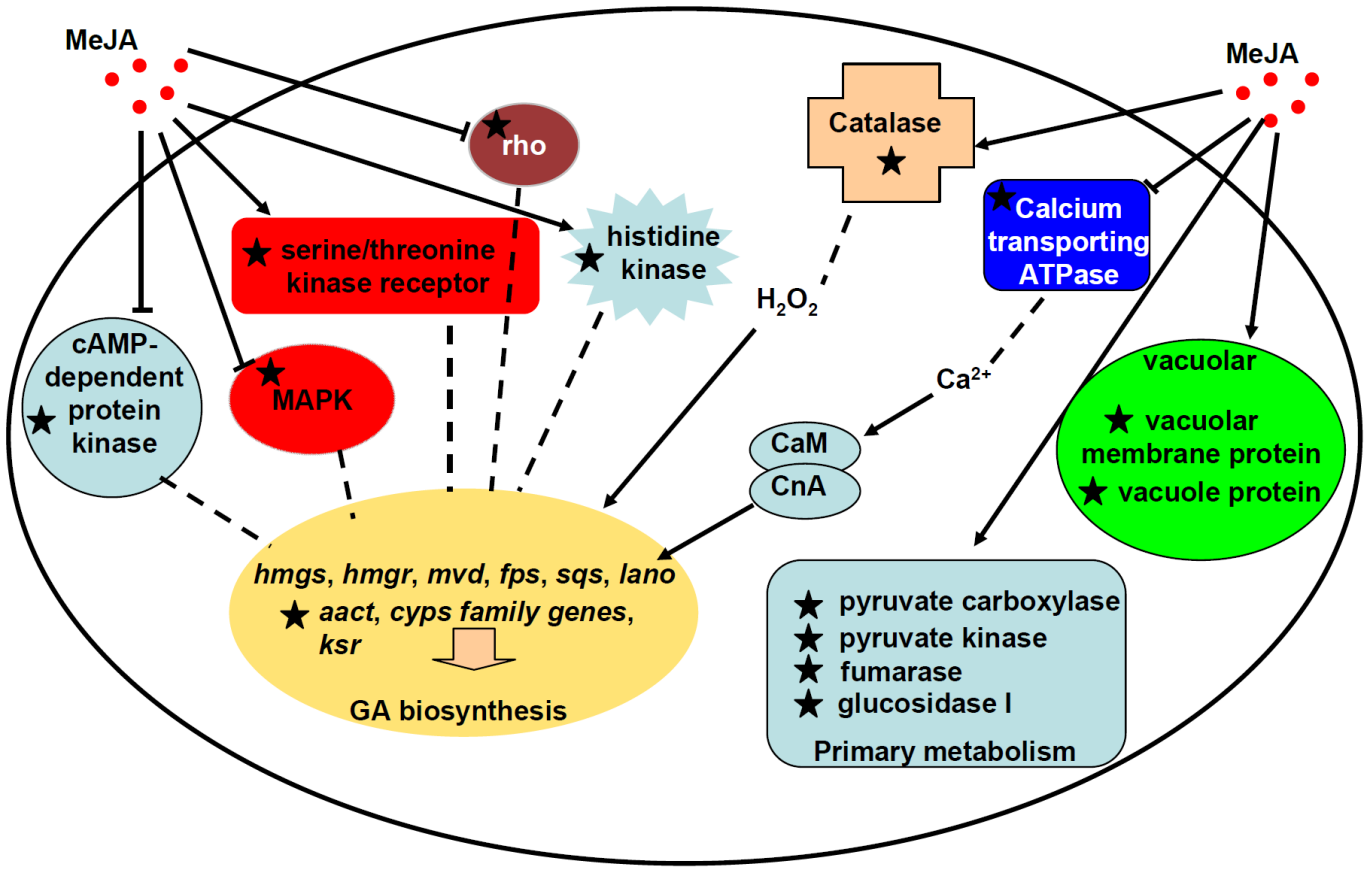
Schematic pathway predicting the role of MeJA induced genes in *G. lucidum*. Integrated pathway map shows the role of MeJA-induced genes involved in GA biosynthesis, primary metabolism, signaling regulation and transcriptional regulation. Dashed lines indicate the probable pathway involved in GA biosynthesis. Solid lines indicate the result supported by cDNA-AFLP and real time PCR. The ESTs from cDNA-AFLP results are indicated in stars.

The biosynthesis of many secondary metabolites is modulated by environmental conditions [36]. Few data are available to elucidate the regulatory mechanisms of the secondary metabolite biosynthesis in response to environmental factors in fungi due to the complex regulatory network and regulatory gene interactions involved [36]–[38]. The same challenge exists for understanding the mechanisms governing the regulation of GA biosynthesis [39], [40]. Despite significant research efforts to understand the influence of environmental factors on the GA biosynthesis [39], the regulatory networks by which environmental factors regulate GA biosynthesis remain unclear. Recent studies on genetically modified GA biosynthesis have focused on the genes restricted to the mevalonate pathway [21], [22]. Our previous study demonstrated that methyl jasmonate can significantly increase both the amount of GA and the transcription levels of known genes in the GA biosynthesis pathway [12]. Additional unknown genes may be involved in the regulatory network of GA biosynthesis. Therefore, the screening of differentially expressed genes during MeJA induction may not only identify novel candidate target genes involved in the regulation of GA biosynthesis but may also provide a new perspective for understanding the regulation of GA biosynthesis (Figure 5).

Among the MeJA up-regulated genes were the acetyl-CoA acetyltransferase gene (TDF113), several members of the cytochrome family (TDF019, TDF047, TDF160, TDF313, TDF322, TDF355, and TDF364), and, most notably, cytochrome P450s (CYPs) (Table 2 and Figure 3). In addition, TDF256, which encodes a 3-keto sterol reductase (*ksr*), was shown to be up-regulated by MeJA induction with both cDNA-AFLP and real-time PCR (Table 2 and Figure 3). In *Saccharomyces cerevisiae*, *ksr* (Erg27p) is required for oxidosqualene cyclase (Erg7p) activity [41], which converts oxidosqualene to lanosterol. Those results suggest that the genes in the mevalonate pathway are up-regulated in response to MeJA induction (Figure 5). This result is consistent with those of previous studies [12], [13] and demonstrates that the genes encoding key enzymes in the mevalonate pathway play an important role in GA biosynthesis. Although not all of the differentially expressed genes in the mevalonate pathway have been detected, the cDNA-AFLP approach is effective for screening differentially expressed genes during MeJA induction.

These results indicate that MeJA induction modulates not only GA biosynthesis-related genes but also related genes in other metabolic pathways, such as glycerol metabolism, pyruvate metabolism, lactate metabolism, sphingolipid metabolism etc. Pyruvate is a precursor of the methylerythritol 4-phosphate (MEP) pathway, and a pyruvate decarboxylase catalyzes the formation of GA [39]. Pyruvate kinase and pyruvate carboxylase are involved in secondary metabolite biosynthesis (Table 2). In this study, TDF243 and TDF323 were identified as pyruvate carboxylase and pyruvate kinase, of which up-regulations were detected using cDNA-AFLP and real time PCR (Table 2 and Figure 3). The result suggested that they were involved in GA biosynthesis. Differential expression of these genes influences a series of metabolic pathways in *G. lucidum* in response to methyl jasmonate (Table S2). This is a complex metabolic regulatory network that includes a series of physiological and biochemical reactions involved in primary and secondary metabolism (Figure 5).

The development stages (fruiting body) and MeJA-treatment improve the GA content [12], [32]. There are 49 genes in both fruiting body EST library and MeJA-induced library, including ERG27-3-keto sterol reductase and cytochrome P450 (Figure 4A and Table S3). Specific expressions of these genes in these two libraries suggest that these genes may not only in response to MeJA but also participate in the developmental process of fruiting body. The investigation of the transcription levels of five genes found that cAMP-dependent protein kinase, aryl-alcohol oxidase, protein kinase activator, ERG27-3-keto sterol reductase, vacuolar membrane protein up-regulated under MeJA treatment and in fruiting body development. The consistent result indicated these five genes may have important roles on the GA biosynthesis. However, there are four genes down-regulated under MeJA treatment and up-regulated in fruiting body development (cAMP-dependent protein kinase, CMGC/MAPK/JNK protein kinase, small monomeric GTPase and nucleotide binding protein) (Figure 3 and Figure 4). Although both MeJA induction and fruiting body stage increased GA content, the regulation mechanism may be difference. These genes may have a variety of physiological functions, especially the regulation of fungal development in development process [42], [43]. Therefore, these four genes probably regulated not only GA biosynthesis in the fruiting body stage, but also the fruiting body development of *Ganoderma lucidum*.

Some signaling factors have been reported to regulate both cell developmental processes and secondary metabolite biosynthesis in filamentous fungi [37]. For example, the small monomeric GTPase rho played an essential role in controlling genes involved in cell polarity, H_2_O_2_ generation, asexual sporulation, and mycotoxin production [42], [44], [45]. The rho-GTPase pathway was associated with cholesterol biosynthesis [46]. In the cDNA-AFLP library, the *G. lucidum* TDF165 gene encodes a protein with sequence similarity to rho (Table 2). The qRT-PCR results confirmed that the rho gene is down-regulated in response to MeJA (Figure 3). Furthermore, the down-regulation of the rho expression level through rho gene silencing can increase the GA content (data not shown). These results suggest that the rho small GTPase pathway represses GA biosynthesis in *G. lucidum*.

A mitogen-activated protein kinase (MAPK) gene (TDF013) was also isolated and characterized from the *G. lucidum* library. The transcription level of the MAPK gene in response to MeJA was 0.20±0.01-fold that of the control. As one of components of the MAPK cascade, a critical signal transduction pathway in eukaryotic organisms, MAPK is essential for regulating growth, differentiation processes and secondary metabolism [47], [48]. Recently, it was reported that the MAPK cascade played an important role in regulating sterigmatocystin biosynthesis [43]. In *G. lucidum*, H_2_O_2_ induced phosphorylation of the proteins Hog-1 and Fus3, which are homologs of the mammalian MAPKs p38 and ERK [49]. In contrast, the mammalian JNK homolog in BCRC 36111 was not detected using a JNK-specific antibody [50]. In this study, the CMGC/MAPK/JNK MAPK is down-regulated after MeJA induction, suggesting that it may play a negative regulatory role in the MeJA signaling pathway (Figure 5).

The sequences of the TDFs that generated significant matches to sequence databases were most commonly genes involved in stress response and cell organization. Two of these TDFs, TDF122, which encodes cell division control protein, and TDF176, which encodes an hsp70-like protein, may also be involved in cell repair and protection against defense responses. TDF 145 and TDF336, which are derived from putative 60S ribosomal protein genes, have also been implicated in responses to oxidative stress, in addition to protein translation for improving carotenoid biosynthesis [51]. Other TDFs, such as *G. lucidum* TDF129 (similar to a catalase), are clearly involved in oxidative stress defense [52]. Previous studies reported that H_2_O_2_ increases GA production [14]. Catalase might be involved in prompt neutralization of H_2_O_2_. The up-regulation of TDF129, a catalase-homologous gene in response to MeJA, indicated that the burst of reactive oxygen species (ROS) triggered by MeJA was most likely involved in GA biosynthesis (Figure 5).

Vacuoles and vesicles are known to sequester secondary metabolites to protect host cells from self-toxicity [53]. Enzymes involved in secondary metabolism, including those for the biosynthesis of cyclosporin, penicillin, and aflatoxin in fungi, are often found in vesicles and vacuoles, [54]–[56]. In *Aspergillus parasiticus*, two enzymatic steps in aflatoxin biosynthesis are completed in vesicles, and these organelles also participate in the compartmentalization and export of the end product, aflatoxin [57]. A vacuole protein gene (TDF360), a vacuolar membrane protein (TDF264) and a COPII-coated vesicle protein gene (TDF327) were also isolated from the *G. lucidum* library (Table 2). The transcription level of the *vmp* gene in response to MeJA induction was 3.10±0.12-fold that of the control (Figure 3).

In conclusion, cDNA-AFLP screening has revealed a number of MeJA-responsive genes in *G. lucidum*. Of the 390 successfully sequenced TDFs, 300 unknown or hypothetical proteins require further characterization to determine whether they are novel MeJA-responsive transcripts. Ninety TDFs were annotated with known functions. MeJA-induced genes expression changes in *G. lucidum* are summarized in a schematic pathway (Figure 5). Experimental data suggests GA biosynthesis relative genes were up-regulated, which included in acetyl-CoA acetyltransferase gene, several members of the cytochrome P450s family, and 3-keto sterol reductase gene (Figure 3 and Table 2). We have previously shown that MeJA induces the expression of six genes (*hmgs*, *hmgr*, *mvd*, *fps*, *sqs*, and *lano*) in the GA biosynthesis pathway [12]. The MeJA induction leads to altered metabolism/energy of *G. lucidum*, which involves changes in primary metabolism and other secondary metabolism, such as glycerol metabolism, pyruvate metabolism, calcium transporting ATPases etc. In previous report found that the calcineurin-signal transduction was significant to GA biosynthesis [58]. The GA biosynthetic genes and the Ca^2+^ sensor were up-regulated with calcium addition. The changes of CMGC/GSK protein kinase, histidine kinase, serine/threonine kinase receptor associated protein, cAMP-dependent protein kinase, rho small monomeric GTPase, MAPK related ESTs signifies a signaling network probably regulated GA biosynthesis under MeJA treatment. But the proper functional characterizations of such genes are still pending. Thus, further characterization of those genes involved in the regulation of GA biosynthesis would lead to an in-depth understanding of GA biosynthesis regulation network.

## Materials and Methods

### Fermentation Conditions and Methyl Jasmonate Elicitation of *G*. *lucidum*

*G. lucidum*, strain HG, was grown at 28°C in potato dextrose agar (PDA) medium. The fermentation conditions of *G. lucidum* were maintained as described [12]. For methyl jasmonate induction, MeJA (Sigma, USA) was dissolved in ethanol and sterilized using a 0.2-µm Supor Membrane Acrodisc Syringe Filter (PALL, USA) before addition to the medium on day 0. The final concentrations of MeJA were 50 and 200 µM. The final ethanol concentration was 2 µL/mL, and equal volumes of ethanol were added to all cultures.

### RNA Extraction Procedure

For each sample, ∼0.5 g of mycelia was collected by filtration from the culture media, dehydrated in liquid nitrogen and stored at –80°C. Total RNA was extracted using an RNA Isolation Kit (Takara, China) and treated with DNase I (Takara, China) according to the manufacturer’s instructions.

### cDNA-AFLP Analysis

The cDNA-AFLP protocol was described previously by Vuylsteke et al. [59]. Double-stranded cDNA was synthesized from 2.5 µg of total RNA using an M-MLV RTase cDNA Synthesis Kit (Takara, China) and an oligo-dT primer (Takara, China).

After pre-amplification, the mixture was diluted 600-fold, and 5 µl was used for selective amplification with each of 64 primer combinations and two selective nucleotides on the MseI primer (Table S4). Touchdown PCR was performed using the following conditions: 2 min of denaturation at 94°C; 13 cycles of 30 s of denaturation at 94°C, 30 s of annealing starting at 65°C and decreasing by 0.7°C per cycle, and 60 s of extension at 72°C; 23 cycles of 30 s of denaturation at 94°C, 30 s of annealing at 56°C, and 60 s of extension at 72°C; and 5 min at 72°C. Selective amplification products were separated on a 6% polyacrylamide gel for 2.5 h at 115 W and 50°C. Images of TDFs were developed by silver staining. TDFs that showed clear differences in intensity were visualized by the Quantity One software Version 4.6 (Bio-Rad, Hercules CA) to identify up-regulated or down-regulated TDFs.

### Sequence Analysis of cDNA-AFLP Fragments

The bands corresponding to differentially expressed genes were excised from the gels with a surgical blade, and the eluted DNA was reamplified using the selective amplification primers and the following PCR conditions: denaturation for 15 min at 94°C; 35 cycles of 40 s of denaturation at 94°C, 60 s of annealing at 56°C, and 40 s of extension at 72°C; and 5 min at 72°C. The quantity of each reamplified band was assessed on a 2% agarose gel, and the DNA was purified from the gel and either sequenced directly using the same primers that were used for the re-amplification or cloned into a pMD-18T vector (Takara, China) and sequenced. Nucleotide and protein sequences were compared to sequences in the available public databases by BLAST sequence alignment. Homology searching was performed against the NCBI databases. The sequences were manually assigned to functional categories based on the analysis of the scientific literature and also with the aid of the information reported for each sequence by the Gene Ontology Consortium [60].

### Real-time RT-PCR Analysis

Real-time RT-PCR was performed on pools of RNA derived from two independent biological experiments. All samples were examined in triplicate. The samples were prepared as described above for the cDNA-AFLP. Total RNA was treated with RNase-free DNase I (Takara, China) according to the manufacturer’s instructions, and 2.5 µg was then used for reverse transcription with Reverse Transcriptase M-MLV (Takara, China). Then, 5 µl of 1∶10 diluted cDNA samples was used as the qRT PCR template with 0.5 µM gene-specific primers and 10 µl SYBR *Premix Ex Taq* II (Takara, China) in a total volume of 20 µl. All samples were examined in triplicate. Experiments were performed in a Realplex2 Systems (Eppendorf, Germany) with the following thermal cycling profile: 95°C for 10 min, followed by 40 cycles of 95°C for 30 s, 55°C for 30 s, and 72°C for 30 s. Each real-time assay was tested in a dissociation protocol to ensure that each amplicon was a single product. The relative quantification of gene expression was performed using the housekeeping gene 18S rRNA [61]. Specific primer pairs were designed for the 25 transcriptionally derived fragments (TDFs) chosen for validation using the Primer 5 software (Table S5). The C_t_ was used to calculate the fold changes (FC) in each gene compared to the expression level detected in the control: FC = 2 ^−ΔΔCt^, where ΔΔC_t_ = (C_t target_−C_t 18s rRNA_) _treated sample_− (C_t target_−C_t 18s rRNA_) _control sample_. Gene expression was evaluated by calculating the difference between the C_t_ of the gene analyzed and the C_t_ of the control 18S rRNA. Post-qRT-PCR calculations analyzing the relative gene expression levels were performed according to the 2^−ΔΔCT^ method described by Livak and Schmittgen [62].

### Statistical Analysis

The significance of samples was determined by analysis of variance, and sample means were separated by the Student’s t-test. Statistical significance was expressed as P<0.05 or P<0.01.

## Supporting Information

Figure S1 **The distribution of 390 TDF on chromosomes in** ***G. lucidum*** **genome.**
(DOC)

Figure S2 **Transcript derived fragments (TDFs) homologies to other known protein found in the** ***G. lucidum*** **genome.**
(DOC)

Figure S3 **Expression patterns of 25 genes in the cDNA-AFLP results.**
(DOC)

Table S1 **Gene Functional Annotations according to Gene Ontology (GO).**
(DOC)

Table S2 **Pathway description of TDFs by searching the KEGG PATHWAY database.**
(DOC)

Table S3 **The genes expressed across MeJA-induction and the different developmental stages.**
(DOC)

Table S4 **Primer sets used for pre-amplified and selective amplified primers.**
(DOC)

Table S5 **Primer sets used for quantitative real-time PCR.**
(DOC)

## References

[1] Zhong JJ, Xiao JH (2009) Secondary metabolites from higher fungi: discovery, bioactivity, and bioproduction. Adv Biochem Eng Biotechnol 113: 79–150. 19475376 10.1007/10_2008_26

[2] Lin ZB (1979) The current pharmacological research on *Ganoderma lucidum* in China. Acta Pharm Sin (in Chinese) 14: 183–192.

[3] Hajjaj H, Mace C, Roberts M, Niederberger P, Fay LB (2005) Effect of 26-oxygenosterols from *Ganoderma lucidum* and their activity as cholesterol synthesis inhibitors. Appl Environ Microbiol 71: 3653–3658. 16000773 10.1128/AEM.71.7.3653-3658.2005 PMC1168986

[4] Joseph S, Sabulal B, George V, Antony KR, Janardhanan KK (2011) Antitumor and anti-inflammatory activities of polysaccharides isolated from *Ganoderma lucidum* . Acta Pharm 61: 335–342. 21945912 10.2478/v10007-011-0030-6

[5] Miyamoto I, Liu J, Shimizu K, Sato M, Kukita A, et al (2009) Regulation of osteoclastogenesis by ganoderic acid DM isolated from *Ganoderma lucidum* . Eur J Pharmacol 602: 1–7. 19026632 10.1016/j.ejphar.2008.11.005

[6] Jedinak A, Thyagarajan-Sahu A, Jiang J, Sliva D (2011) Ganodermanontriol, a lanostanoid triterpene from *Ganoderma lucidum*, suppresses growth of colon cancer cells through ss-catenin signaling. Int J Oncol 38: 761–767. 21225227 10.3892/ijo.2011.898

[7] Kimura Y, Taniguchi M, Baba K (2002) Antitumor and antimetastatic effects on liver of triterpenoid fractions of *Ganoderma lucidum*: mechanism of action and isolation of an active substance. Anticancer Res 22: 3309–3318. 12530080

[8] Li N, Liu XH, Zhou J, Li YX, Zhao MW (2006) Analysis of Influence of Environmental Conditions on Ganoderic Acid Content in *Ganoderma lucidum* Using Orthogonal Design. J Microbiol Biotechnol 16: 1940–1946.

[9] Tang YJ, Zhang W, Zhong JJ (2009) Performance analyses of a pH-shift and DOT-shift integrated fed-batch fermentation process for the production of ganoderic acid and Ganoderma polysaccharides by medicinal mushroom *Ganoderma lucidum* . Bioresour Technol 100: 1852–1859. 19010665 10.1016/j.biortech.2008.10.005

[10] Mach-Aigner AR, Pucher ME, Mach RL (2010) D-Xylose as a repressor or inducer of xylanase expression in *Hypocrea jecorina* (Trichoderma reesei). Appl Environ Microbiol 76: 1770–1776. 20097821 10.1128/AEM.02746-09 PMC2838004

[11] Zhang BB, Cheung PC (2011) Use of stimulatory agents to enhance the production of bioactive exopolysaccharide from pleurotus tuber-regium by submerged fermentation. J Agric Food Chem 59: 1210–1216. 21280626 10.1021/jf104425w

[12] Ren A, Qin L, Shi L, Dong X, Mu da S, et al (2010) Methyl jasmonate induces ganoderic acid biosynthesis in the basidiomycetous fungus *Ganoderma lucidum* . Bioresour Technol 101: 6785–6790. 20395130 10.1016/j.biortech.2010.03.118

[13] Liang CX, Li YB, Xu JW, Wang JL, Miao XL, et al (2010) Enhanced biosynthetic gene expressions and production of ganoderic acids in static liquid culture of *Ganoderma lucidum* under phenobarbital induction. Appl Microbiol Biotechnol 86: 1367–1374. 20077112 10.1007/s00253-009-2415-8

[14] Zhang WX, Tang YJ, Zhong JJ (2010) Impact of oxygen level in gaseous phase on gene transcription and ganoderic acid biosynthesis in liquid static cultures of *Ganoderma lucidum* . Bioprocess Biosyst Eng 33: 683–690. 19809834 10.1007/s00449-009-0379-9

[15] Shiao MS (1992) Triterpenoid natural products in the fungus *Ganoderma lucidum* . J Chin Chem Soc 39: 669–674.

[16] Hirotani M, Asaka I, Furuya T (1990) Investigation of the biosynthesis of 3α-hydroxy triterpenoids, ganoderic acids T and S, by application of a feeding experiment using [1, 2–^13^C_2_]acetate. J Chem Soc Perkin Trans 1: 2751–2754.

[17] Zhao MW, Liang WQ, Zhang DB, Wang N, Wang CG, et al (2007) Cloning and characterization of squalene synthase (SQS) gene from *Ganoderma lucidum* . J Microbiol Biotechnol 17: 1106–1112. 18051320

[18] Ding YX, Ou-Yang X, Shang CH, Ren A, Shi L, et al (2008) Molecular cloning, characterization, and differential expression of a farnesyl-diphosphate synthase gene from the basidiomycetous fungus *Ganoderma lucidum* . Biosci Biotechnol Biochem 72: 1571–1579. 18540102 10.1271/bbb.80067

[19] Shang CH, Zhu F, Li N, Ou-Yang X, Shi L, et al (2008) Cloning and characterization of a gene encoding HMG-CoA reductase from *Ganoderma lucidum* and its functional identification in yeast. Biosci Biotechnol Biochem 72: 1333–1339. 18460810 10.1271/bbb.80011

[20] Shang CH, Shi L, Ren A, Qin L, Zhao MW (2010) Molecular cloning, characterization, and differential expression of a lanosterol synthase gene from *Ganoderma lucidum* . Biosci Biotechnol Biochem 74: 974–978. 20460708 10.1271/bbb.90833

[21] Shi L, Qin L, Xu Y, Ren A, Fang X, et al (2012) Molecular cloning, characterization, and function analysis of a mevalonate pyrophosphate decarboxylase gene from *Ganoderma lucidum* . Mol Biol Rep 39: 6149–6159. 22203490 10.1007/s11033-011-1431-9

[22] Xu JW, Xu YN, Zhong JJ (2012) Enhancement of Ganoderic Acid Accumulation by Overexpression of an N-Terminally Truncated 3-Hydroxy-3-Methylglutaryl Coenzyme A Reductase Gene in the Basidiomycete *Ganoderma lucidum* . Appl Environ Microbiol 78: 7968–7976. 22941092 10.1128/AEM.01263-12 PMC3485969

[23] Ren A, Ouyang X, Shi L, Jiang AL, Mu DS, et al (2013) Molecular characterization and expression analysis of GlHMGS, a gene encoding hydroxymethylglutaryl-CoA synthase from *Ganoderma lucidum* (Ling-zhi) in ganoderic acid biosynthesis pathway. World J Microbiol Biotechnol 29: 523–531. 23138457 10.1007/s11274-012-1206-z

[24] Creelman RA, Tierney ML, Mullet JE (1992) Jasmonic acid/methyl jasmonate accumulate in wounded soybean hypocotyls and modulate wound gene expression. Proc Natl Acad Sci U S A 89: 4938–4941. 1594598 10.1073/pnas.89.11.4938 PMC49203

[25] Choi D, Bostock RM, Avdiushko S, Hildebrand DF (1994) Lipid-derived signals that discriminate wound- and pathogen-responsive isoprenoid pathways in plants: methyl jasmonate and the fungal elicitor arachidonic acid induce different 3-hydroxy-3-methylglutaryl-coenzyme A reductase genes and antimicrobial isoprenoids in *Solanum tuberosum* L. Proc Natl Acad Sci U S A. 91: 2329–2333. 10.1073/pnas.91.6.2329 PMC43364 11607466

[26] Oh SY, Kim JH, Park MJ, Kim SM, Yoon CS, et al (2005) Induction of heat shock protein 72 in C6 glioma cells by methyl jasmonate through ROS-dependent heat shock factor 1 activation. Int J Mol Med 16: 833–839. 16211252

[27] Raina SK, Wankhede DP, Jaggi M, Singh P, Jalmi SK, et al (2012) CrMPK3, a mitogen activated protein kinase from *Catharanthus roseus* and its possible role in stress induced biosynthesis of monoterpenoid indole alkaloids. BMC Plant Biol 12: 134. 22871174 10.1186/1471-2229-12-134 PMC3487899

[28] Munemasa S, Hossain MA, Nakamura Y, Mori IC, Murata Y (2011) The Arabidopsis calcium-dependent protein kinase, CPK6, functions as a positive regulator of methyl jasmonate signaling in guard cells. Plant Physiol 155: 553–561. 20978156 10.1104/pp.110.162750 PMC3075756

[29] Yao HJ, Tian SP (2005) Effects of a biocontrol agent and methyl jasmonate on postharvest diseases of peach fruit and the possible mechanisms involved. J Appl Microbiol 98: 941–950. 15752341 10.1111/j.1365-2672.2004.02531.x

[30] Meimaroglou DM, Galanopoulou D, Markaki P (2009) Study of the Effect of Methyl Jasmonate Concentration on Aflatoxin B(1) Biosynthesis by *Aspergillus parasiticus* in Yeast Extract Sucrose Medium. Int J Microbiol 2009: 842626. 20016812 10.1155/2009/842626 PMC2789378

[31] Vergopoulou S, Galanopoulou D, Markaki P (2001) Methyl jasmonate stimulates aflatoxin B1 biosynthesis by *Aspergillus parasiticus* . J Agric Food Chem 49: 3494–3498. 11453798 10.1021/jf010074+

[32] Chen S, Xu J, Liu C, Zhu Y, Nelson DR, et al (2012) Genome sequence of the model medicinal mushroom *Ganoderma lucidum* . Nat Commun 3: 913. 22735441 10.1038/ncomms1923 PMC3621433

[33] Liu D, Gong J, Dai W, Kang X, Huang Z, et al (2012) The genome of *Ganoderma lucidum* provides insights into triterpenes biosynthesis and wood degradation. PLoS One 7: e36146. 22567134 10.1371/journal.pone.0036146 PMC3342255

[34] Conesa A, Gotz S, Garcia-Gomez JM, Terol J, Talon M, et al (2005) Blast2GO: a universal tool for annotation, visualization and analysis in functional genomics research. Bioinformatics 21: 3674–3676. 16081474 10.1093/bioinformatics/bti610

[35] Yonekura-Sakakibara K, Tohge T, Matsuda F, Nakabayashi R, Takayama H, et al (2008) Comprehensive flavonol profiling and transcriptome coexpression analysis leading to decoding gene-metabolite correlations in *Arabidopsis* . Plant Cell 20: 2160–2176. 18757557 10.1105/tpc.108.058040 PMC2553606

[36] Yu JH, Keller N (2005) Regulation of secondary metabolism in filamentous fungi. Annu Rev Phytopathol 43: 437–458. 16078891 10.1146/annurev.phyto.43.040204.140214

[37] Calvo AM, Wilson RA, Bok JW, Keller NP (2002) Relationship between secondary metabolism and fungal development. Microbiol Mol Biol Rev 66: 447–459. 12208999 10.1128/MMBR.66.3.447-459.2002 PMC120793

[38] Yin WB, Amaike S, Wohlbach DJ, Gasch AP, Chiang YM, et al (2012) An Aspergillus nidulans bZIP response pathway hardwired for defensive secondary metabolism operates through aflR. Mol Microbiol 83: 1024–1034. 22283524 10.1111/j.1365-2958.2012.07986.x PMC3288630

[39] Shi L, Ren A, Mu D, Zhao M (2010) Current progress in the study on biosynthesis and regulation of ganoderic acids. Appl Microbiol Biotechnol 88: 1243–1251. 20859739 10.1007/s00253-010-2871-1

[40] Yu GJ, Wang M, Huang J, Yin YL, Chen YJ, et al (2012) Deep insight into the *Ganoderma lucidum* by comprehensive analysis of its transcriptome. PLoS One 7: e44031. 22952861 10.1371/journal.pone.0044031 PMC3428325

[41] Mo C, Milla P, Athenstaedt K, Ott R, Balliano G, et al (2003) In yeast sterol biosynthesis the 3-keto reductase protein (Erg27p) is required for oxidosqualene cyclase (Erg7p) activity. Biochim Biophys Acta 1633: 68–74. 12842197 10.1016/s1388-1981(03)00088-x

[42] Zheng W, Zhao Z, Chen J, Liu W, Ke H, et al (2009) A Cdc42 ortholog is required for penetration and virulence of *Magnaporthe grisea* . Fungal Genet Biol 46: 450–460. 19298860 10.1016/j.fgb.2009.03.005

[43] Bayram O, Bayram OS, Ahmed YL, Maruyama J, Valerius O, et al (2012) The Aspergillus nidulans MAPK module AnSte11-Ste50-Ste7-Fus3 controls development and secondary metabolism. PLoS Genet 8: e1002816. 22829779 10.1371/journal.pgen.1002816 PMC3400554

[44] Raudaskoski M, Kothe E, Fowler TJ, Jung EM, Horton JS (2012) Ras and Rho small G proteins: insights from the *Schizophyllum commune* genome sequence and comparisons to other fungi. Biotechnol Genet Eng Rev 28: 61–100. 22616482 10.5661/bger-28-61

[45] Song J, Li J, Lulla A, Evers BM, Chung DH (2006) Protein kinase D protects against oxidative stress-induced intestinal epithelial cell injury via Rho/ROK/PKC-delta pathway activation. Am J Physiol Cell Physiol 290: C1469–1476. 16421204 10.1152/ajpcell.00486.2005 PMC2613753

[46] Quetglas JI, Hernaez B, Galindo I, Munoz-Moreno R, Cuesta-Geijo MA, et al (2012) Small rho GTPases and cholesterol biosynthetic pathway intermediates in African swine fever virus infection. J Virol 86: 1758–1767. 22114329 10.1128/JVI.05666-11 PMC3264358

[47] Nishida E, Gotoh Y (1993) The MAP kinase cascade is essential for diverse signal transduction pathways. Trends Biochem Sci 18: 128–131. 8388132 10.1016/0968-0004(93)90019-j

[48] Atoui A, Bao DP, Kaur N, Grayburn WS, Calvo AM (2008) *Aspergillus nidulans* natural product biosynthesis is regulated by mpkB, a putative pheromone response mitogen-activated protein kinase. Appl Environ Microbiol 74: 3596–3600. 18378656 10.1128/AEM.02842-07 PMC2423048

[49] You BJ, Lee MH, Tien N, Lee MS, Hsieh HC, et al (2013) A Novel Approach to Enhancing Ganoderic Acid Production by *Ganoderma lucidum* Using Apoptosis Induction. PLoS One 8: e53616. 23326470 10.1371/journal.pone.0053616 PMC3542374

[50] You B-J, Chang W-T, Chung K-R, Kuo Y-H, Yang C-S, et al (2012) Effect of solid-medium coupled with reactive oxygen species on ganoderic acid biosynthesis and MAP kinase phosphorylation in *Ganoderma lucidum* . Food Research International 49: 634–640.

[51] Mendez-Alvarez S, Rufenacht K, Eggen RI (2000) The oxidative stress-sensitive yap1 null strain of Saccharomyces cerevisiae becomes resistant due to increased carotenoid levels upon the introduction of the *Chlamydomonas reinhardtii* cDNA, coding for the 60S ribosomal protein L10a. Biochem Biophys Res Commun 267: 953–959. 10673398 10.1006/bbrc.1999.2070

[52] Rochat T, Gratadoux JJ, Gruss A, Corthier G, Maguin E, et al (2006) Production of a heterologous nonheme catalase by Lactobacillus casei: an efficient tool for removal of H_2_O_2_ and protection of *Lactobacillus bulgaricus* from oxidative stress in milk. Appl Environ Microbiol 72: 5143–5149. 16885258 10.1128/AEM.00482-06 PMC1538758

[53] Sirikantaramas S, Yamazaki M, Saito K (2008) Mechanisms of resistance to self-produced toxic secondary metabolites in plants. Phytochemistry Reviews 7: 467–477.

[54] Hoppert M, Gentzsch C, Schorgendorfer K (2001) Structure and localization of cyclosporin synthetase, the key enzyme of cyclosporin biosynthesis in *Tolypocladium inflatum* . Arch Microbiol 176: 285–293. 11685373 10.1007/s002030100324

[55] Lendenfeld T, Ghali D, Wolschek M, Kubicek-Pranz EM, Kubicek CP (1993) Subcellular compartmentation of penicillin biosynthesis in *Penicillium chrysogenum*. The amino acid precursors are derived from the vacuole. J Biol Chem 268: 665–671. 8416970

[56] Hong SY, Linz JE (2008) Functional expression and subcellular localization of the aflatoxin pathway enzyme Ver-1 fused to enhanced green fluorescent protein. Appl Environ Microbiol 74: 6385–6396. 18757582 10.1128/AEM.01185-08 PMC2570292

[57] Chanda A, Roze LV, Kang S, Artymovich KA, Hicks GR, et al (2009) A key role for vesicles in fungal secondary metabolism. Proc Natl Acad Sci U S A 106: 19533–19538. 19889978 10.1073/pnas.0907416106 PMC2773199

[58] Xu YN, Zhong JJ (2012) Impacts of calcium signal transduction on the fermentation production of antitumor ganoderic acids by medicinal mushroom *Ganoderma lucidum* . Biotechnol Adv 30: 1301–1308. 22036615 10.1016/j.biotechadv.2011.10.001

[59] Vuylsteke M, Peleman JD, van Eijk MJ (2007) AFLP-based transcript profiling (cDNA-AFLP) for genome-wide expression analysis. Nat Protoc 2: 1399–1413. 17545977 10.1038/nprot.2007.174

[60] Ashburner M, Ball CA, Blake JA, Botstein D, Butler H, et al (2000) Gene ontology: tool for the unification of biology. The Gene Ontology Consortium. Nat Genet 25: 25–29. 10802651 10.1038/75556 PMC3037419

[61] Mu D, Shi L, Ren A, Li M, Wu F, et al (2012) The development and application of a multiple gene co-silencing system using endogenous URA3 as a reporter gene in *Ganoderma lucidum* . PLoS One 7: e43737. 22937087 10.1371/journal.pone.0043737 PMC3427163

[62] Livak KJ, Schmittgen TD (2001) Analysis of relative gene expression data using real-time quantitative PCR and the 2(-Delta Delta C(T)) Method. Methods 25: 402–408. 11846609 10.1006/meth.2001.1262

