# Supplementary material for: Profiling and Quantifying Differential Gene Transcription Provide Insights into Ganoderic Acid Biosynthesis in Ganoderma lucidum in Response to Methyl Jasmonate
Source: PLoS One. 2013 Jun 7;8(6):e65027. doi: 10.1371/journal.pone.0065027 (PMC3676390; doi:10.1371/journal.pone.0065027)
Supplement: Figure S1 — The distribution of 390 TDF on chromosomes in G. lucidum genome. (DOC) [file pone.0065027.s001.doc]

Ang Ren, et.al., supplemental material file: Figure S1


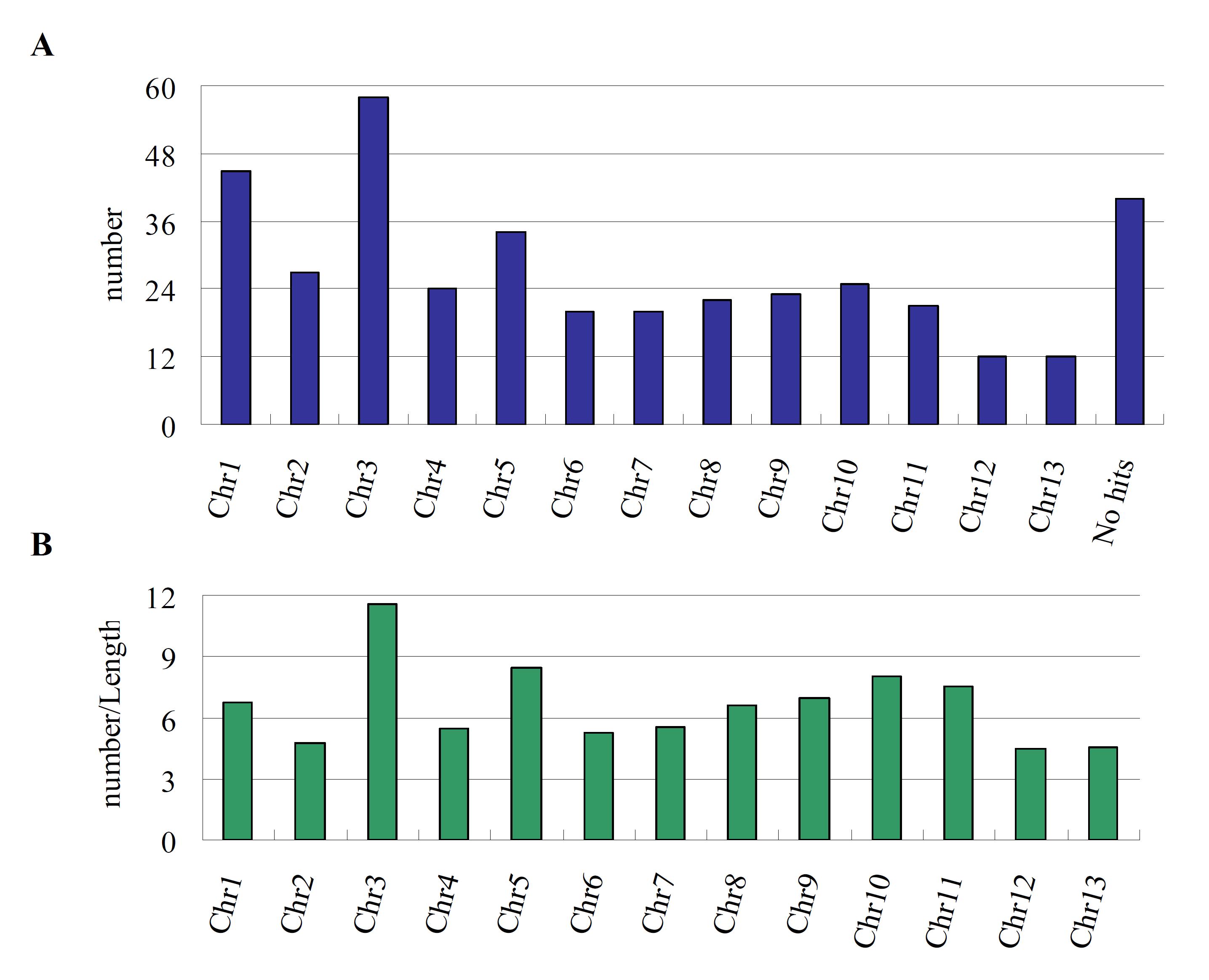


Figure S1. The distribution of 390 TDF on chromosomes in *G. lucidum* genome.

A, the number of TDFs on the each chromosome (Chr1-Chr13). No hits means that several TDFs were not detected on the *G*. *lucidum* genome. B, the TDFs density in *G*. *lucidum* genome.
