# Supplementary material for: Profiling and Quantifying Differential Gene Transcription Provide Insights into Ganoderic Acid Biosynthesis in Ganoderma lucidum in Response to Methyl Jasmonate
Source: PLoS One. 2013 Jun 7;8(6):e65027. doi: 10.1371/journal.pone.0065027 (PMC3676390; doi:10.1371/journal.pone.0065027)
Supplement: Figure S2 — Transcript derived fragments (TDFs) homologies to other known protein found in the G. lucidum genome. (DOC) [file pone.0065027.s002.doc]

Ang Ren, et.al., supplemental material file: Figure S2


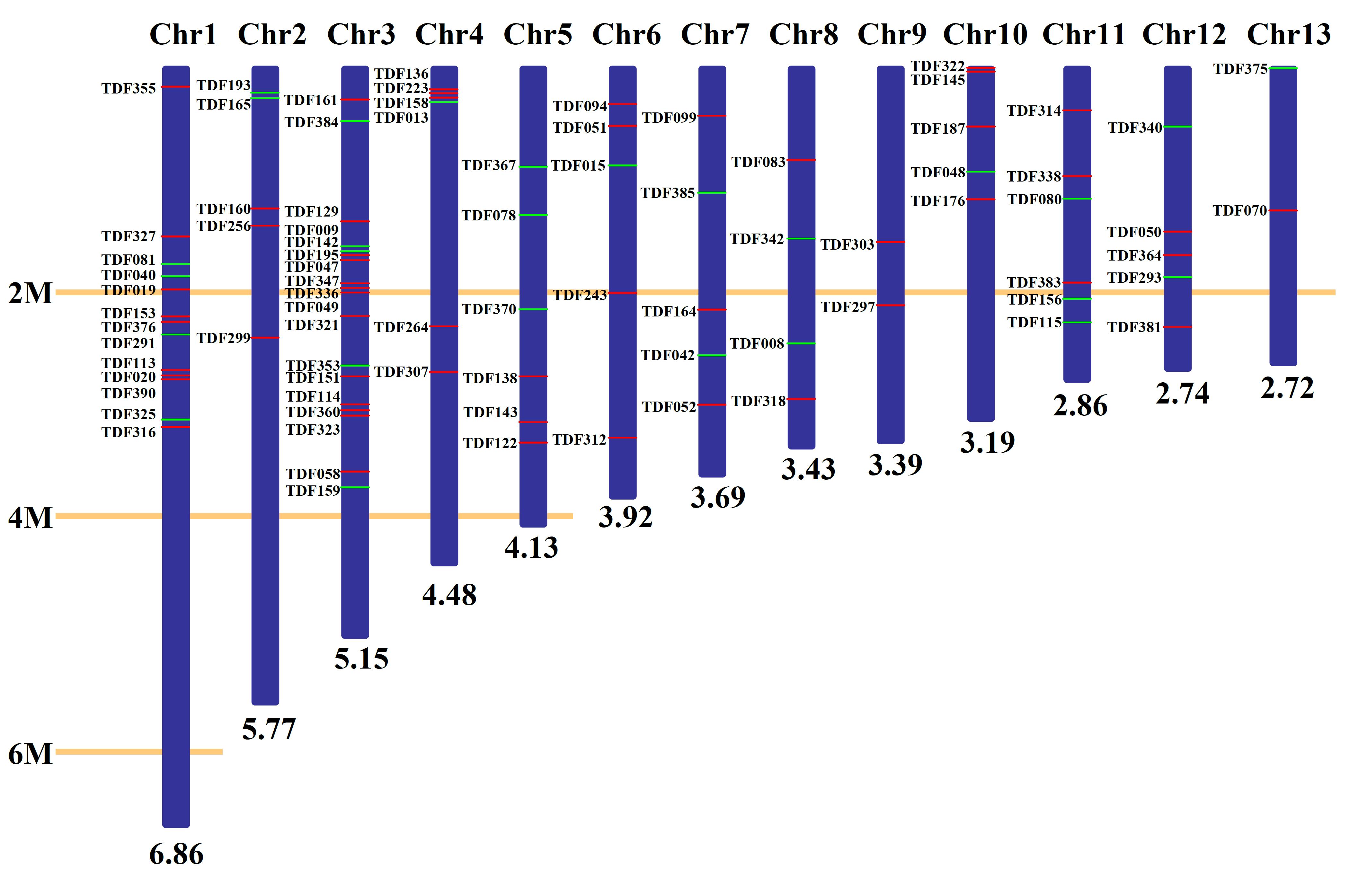


Figure S2. Transcript derived fragments (TDFs) homologies to other known protein found in the *G. lucidum* genome.

The TDFs homologies to other known protein are represented by lines on the chromosomes. The colours of the lines indicate whether the TDFs are up-regulated (red) or down-regulated (green). The TDF numbers are shown to the left. The chromosome numbers are shown at the top. The chromosome sizes are shown at the bottom.
