# Supplementary material for: Profiling and Quantifying Differential Gene Transcription Provide Insights into Ganoderic Acid Biosynthesis in Ganoderma lucidum in Response to Methyl Jasmonate
Source: PLoS One. 2013 Jun 7;8(6):e65027. doi: 10.1371/journal.pone.0065027 (PMC3676390; doi:10.1371/journal.pone.0065027)
Supplement: Figure S3 — Expression patterns of 25 genes in the cDNA-AFLP results. (DOC) [file pone.0065027.s003.doc]

Ang Ren, et.al., supplemental material file: Figure S3


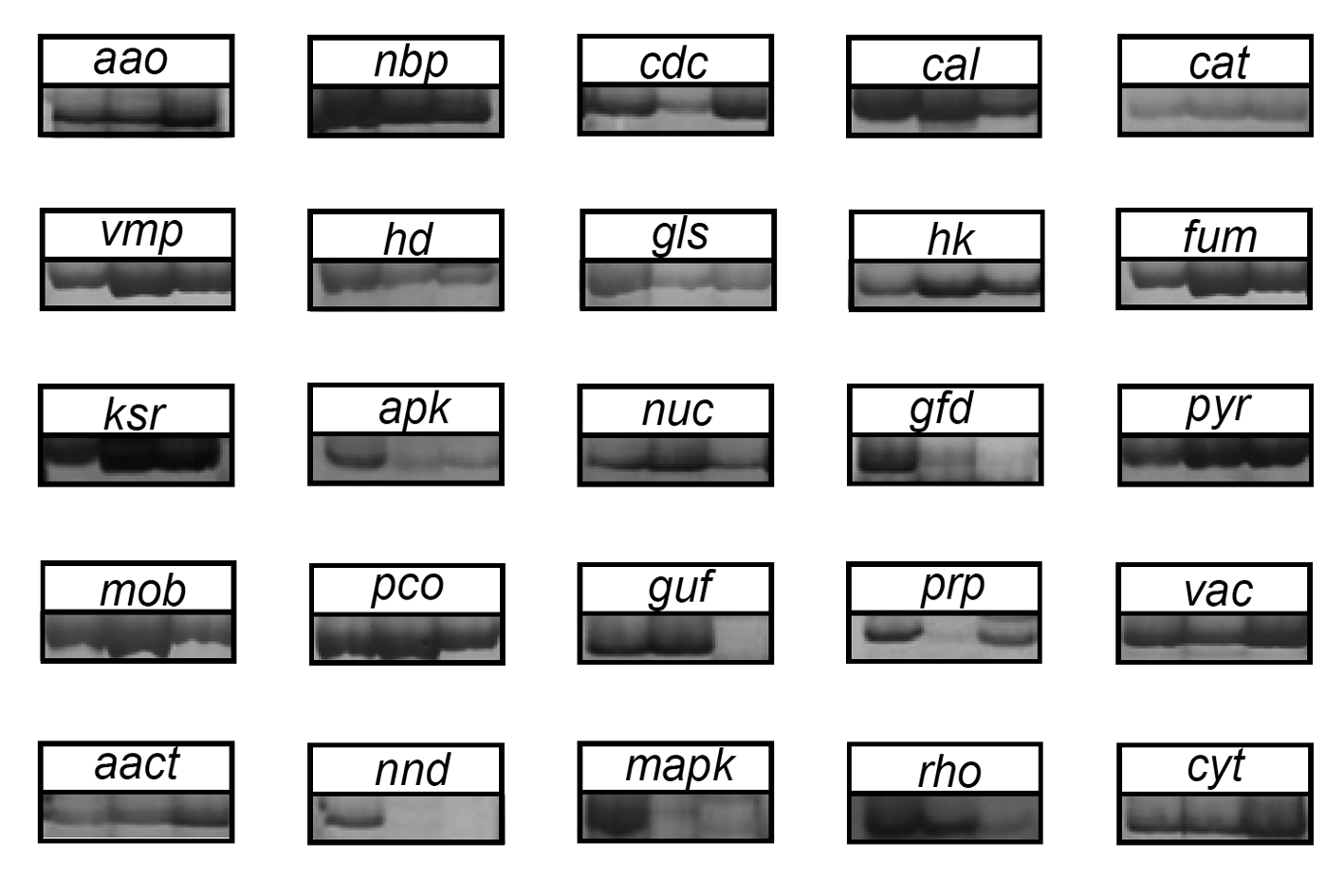


**Figure S3. Expression patterns of 25 genes in the cDNA-AFLP results.** This figure shows the expression patterns of 25 genes under MeJA treatment. The upper layer is the abbreviation of each gene, of which full name is in the Figure 3 legends. The lower layer is the expression patterns of each gene. The left, middle and right lanes are the expression patterns of 25 genes treated with 0, 50 and 200 μM MeJA, respectively.
