# Supplementary material for: Profiling and Quantifying Differential Gene Transcription Provide Insights into Ganoderic Acid Biosynthesis in Ganoderma lucidum in Response to Methyl Jasmonate
Source: PLoS One. 2013 Jun 7;8(6):e65027. doi: 10.1371/journal.pone.0065027 (PMC3676390; doi:10.1371/journal.pone.0065027)
Supplement: Table S1 — Gene Functional Annotations according to Gene Ontology (GO). (DOC) [file pone.0065027.s004.doc]

Ang Ren, *et.al*., supplemental material file: Table S1

Table S1 Gene Functional Annotations according to Gene Ontology (GO)

| Seq. Name | Functional Annotations | | | |
| --- | --- | --- | --- | --- |
| TDF006 | GO:0003824:catalytic activity | GO:0015926:glucosidase activity | GO:0043169:cation binding | GO:0005975:carbohydrate metabolic process |
| TDF008 | GO:0005622:intracellular | GO:0005840:ribosome | GO:0003735:structural constituent of ribosome | GO:0006412:translation |
| TDF013 | GO:0004672:protein kinase activity | GO:0004674:protein serine/threonine kinase activity | GO:0005524:ATP binding | GO:0006468:protein amino acid phosphorylation |
| TDF015 | GO:0008270:zinc ion binding | GO:0016811:hydrolase activity, acting on carbon-nitrogen (but not peptide) bonds, in linear amides | GO:0070403:NAD binding | GO:0006342:chromatin silencing |
|  | GO:0006476:protein amino acid deacetylation | GO:0045449:regulation of transcription |  |  |
| TDF019 | GO:0020037:heme binding |  |  |  |
| TDF020 | GO:0003677:DNA binding | GO:0003700:transcription factor activity | GO:0006355:regulation of transcription, DNA-dependent |  |
| TDF040 | GO:0004672:protein kinase activity | GO:0004674:protein serine/threonine kinase activity | GO:0005524:ATP binding | GO:0006468:protein amino acid phosphorylation |
| TDF042 | GO:0005622:intracellular | GO:0005634:nucleus | GO:0005488:binding | GO:0000398:nuclear mRNA splicing, via spliceosome |
|  | GO:0006396:RNA processing |  |  |  |
| TDF047 | GO:0003824:catalytic activity | GO:0010181:FMN binding | GO:0016491:oxidoreductase activity | GO:0020037:heme binding |
|  | GO:0008152:metabolic process | GO:0055114:oxidation reduction |  |  |
| TDF048 | GO:0005622:intracellular | GO:0006810:transport |  |  |
| TDF049 | GO:0005737:cytoplasm | GO:0003746:translation elongation factor activity | GO:0003924:GTPase activity | GO:0004672:protein kinase activity |
|  | GO:0004674:protein serine/threonine kinase activity | GO:0005524:ATP binding | GO:0005525:GTP binding | GO:0006414:translational elongation |
|  | GO:0006468:protein amino acid phosphorylation |  |  |  |
| TDF050 | GO:0000785:chromatin | GO:0005634:nucleus | GO:0003682:chromatin binding | GO:0016491:oxidoreductase activity |
|  | GO:0006333:chromatin assembly or disassembly |  |  |  |
| TDF051 | GO:0016020:membrane | GO:0000155:two-component sensor activity | GO:0000156:two-component response regulator activity | GO:0004673:protein histidine kinase activity |
|  | GO:0004871:signal transducer activity | GO:0005524:ATP binding | GO:0016772:transferase activity, transferring phosphorus-containing groups | GO:0000160:two-component signal transduction system (phosphorelay) |
|  | GO:0006355:regulation of transcription, DNA-dependent | GO:0007165:signal transduction | GO:0016310:phosphorylation | GO:0018106:peptidyl-histidine phosphorylation |
|  | GO:0007165:signal transduction | GO:0016310:phosphorylation | GO:0018106:peptidyl-histidine phosphorylation |  |
| TDF052 | GO:0005515:protein binding |  |  |  |
| TDF058 | GO:0005789:endoplasmic reticulum membrane | GO:0004622:lysophospholipase activity | GO:0006629:lipid metabolic process | GO:0008152:metabolic process |
|  | GO:0046470:phosphatidylcholine metabolic process |  |  |  |
| TDF078 | GO:0016020:membrane | GO:0005215:transporter activity | GO:0006810:transport |  |
| TDF080 | GO:0000148:1,3-beta-glucan synthase complex | GO:0016020:membrane | GO:0003843:1,3-beta-glucan synthase activity | GO:0006075:1,3-beta-glucan biosynthetic process |
| TDF081 | GO:0005634:nucleus | GO:0003677:DNA binding | GO:0003887:DNA-directed DNA polymerase activity | GO:0006260:DNA replication |
|  | GO:0006261:DNA-dependent DNA replication |  |  |  |
| TDF083 | GO:0046872:metal ion binding | GO:0030001:metal ion transport |  |  |
| TDF094 | GO:0003723:RNA binding | GO:0003899:DNA-directed RNA polymerase activity | GO:0006350:transcription |  |
| TDF096 | GO:0016614:oxidoreductase activity | GO:0050660:FAD binding | GO:0006066:alcohol metabolic process |  |
| TDF099 | GO:0000287:magnesium ion binding | GO:0003824:catalytic activity | GO:0030976:thiamin pyrophosphate binding |  |
| TDF113 | GO:0003824:catalytic activity | GO:0016747:transferase activity, transferring acyl groups other than amino-acyl groups | GO:0008152:metabolic process |  |
| TDF114 | GO:0003743:translation initiation factor activity | GO:0006413:translational initiation |  |  |
| TDF115 | GO:0003824:catalytic activity | GO:0004077:biotin-[acetyl-CoA-carboxylase] ligase activity | GO:0006464:protein modification process |  |
| TDF122 | GO:0005680:anaphase-promoting complex | GO:0005488:binding | GO:0030071:regulation of mitotic metaphase/anaphase transition |  |
| TDF129 | GO:0004096:catalase activity | GO:0005506:iron ion binding | GO:0020037:heme binding | GO:0006979:response to oxidative stress |
|  | GO:0055114:oxidation reduction |  |  |  |
| TDF136 | GO:0005622:intracellular | GO:0005515:protein binding | GO:0016881:acid-amino acid ligase activity | GO:0006464:protein modification process |
| TDF138 | GO:0003824:catalytic activity | GO:0004049:anthranilate synthase activity | GO:0004425:indole-3-glycerol-phosphate synthase activity | GO:0004640:phosphoribosylanthranilate isomerase activity |
|  | GO:0006541:glutamine metabolic process | GO:0006568:tryptophan metabolic process | GO:0008152:metabolic process | GO:0009058:biosynthetic process |
| TDF142 | GO:0003824:catalytic activity | GO:0005488:binding | GO:0016491:oxidoreductase activity | GO:0008152:metabolic process |
|  | GO:0055114:oxidation reduction |  |  |  |
| TDF143 | GO:0047547:2-methylcitrate dehydratase activity | GO:0051537:2 iron, 2 sulfur cluster binding | GO:0019543:propionate catabolic process | GO:0019629:propionate catabolic process, 2-methylcitrate cycle |
| TDF145 | GO:0005622:intracellular | GO:0005840:ribosome | GO:0003735:structural constituent of ribosome | GO:0006412:translation |
| TDF151 | GO:0016020:membrane | GO:0005515:protein binding | GO:0016192:vesicle-mediated transport |  |
| TDF153 | GO:0045239:tricarboxylic acid cycle enzyme complex | GO:0003824:catalytic activity | GO:0004333:fumarate hydratase activity | GO:0016829:lyase activity |
|  | GO:0006099:tricarboxylic acid cycle | GO:0006106:fumarate metabolic process |  |  |
| TDF156 | GO:0003676:nucleic acid binding |  |  |  |
| TDF158 | GO:0004672:protein kinase activity | GO:0004674:protein serine/threonine kinase activity | GO:0005524:ATP binding | GO:0006468:protein amino acid phosphorylation |
| TDF159 | GO:0030904:retromer complex | GO:0007034:vacuolar transport |  |  |
| TDF160 | GO:0004497:monooxygenase activity | GO:0005506:iron ion binding | GO:0009055:electron carrier activity | GO:0020037:heme binding |
|  | GO:0055114:oxidation reduction |  |  |  |
| TDF161 | GO:0004553:hydrolase activity, hydrolyzing O-glycosyl compounds | GO:0005975:carbohydrate metabolic process |  |  |
| TDF164 | GO:0004222:metalloendopeptidase activity | GO:0006508:proteolysis |  |  |
| TDF165 | GO:0005622:intracellular | GO:0005525:GTP binding | GO:0007264:small GTPase mediated signal transduction | GO:0015031:protein transport |
| TDF176 | GO:0031072:heat shock protein binding |  |  |  |
| TDF187 | GO:0055085:transmembrane transport |  |  |  |
| TDF193 | GO:0005622:intracellular | GO:0005525:GTP binding | GO:0007264:small GTPase mediated signal transduction | GO:0015031:protein transport |
| TDF195 | GO:0045239:tricarboxylic acid cycle enzyme complex | GO:0003824:catalytic activity | GO:0004333:fumarate hydratase activity | GO:0016829:lyase activity |
|  | GO:0006099:tricarboxylic acid cycle | GO:0006106:fumarate metabolic process |  |  |
| TDF223 | GO:0004553:hydrolase activity, hydrolyzing O-glycosyl compounds | GO:0005975:carbohydrate metabolic process |  |  |
| TDF243 | GO:0005737:cytoplasm | GO:0003824:catalytic activity | GO:0004736:pyruvate carboxylase activity | GO:0005524:ATP binding |
|  | GO:0009374:biotin binding | GO:0016874:ligase activity | GO:0006094:gluconeogenesis | GO:0008152:metabolic process |
| TDF256 | GO:0016491:oxidoreductase activity | GO:0008152:metabolic process |  |  |
| TDF264 | GO:0030904:retromer complex | GO:0007034:vacuolar transport |  |  |
| TDF291 | GO:0003824:catalytic activity | GO:0005488:binding | GO:0008270:zinc ion binding | GO:0016491:oxidoreductase activity |
|  | GO:0051903:S-(hydroxymethyl)glutathione dehydrogenase activity | GO:0006069:ethanol oxidation | GO:0008152:metabolic process | GO:0055114:oxidation reduction |
| TDF293 | GO:0004407:histone deacetylase activity | GO:0016575:histone deacetylation |  |  |
| TDF297 | GO:0006457:protein folding |  |  |  |
| TDF299 | GO:0000786:nucleosome | GO:0005634:nucleus | GO:0003677:DNA binding | GO:0006334:nucleosome assembly |
| TDF303 | GO:0015629:actin cytoskeleton | GO:0003779:actin binding | GO:0007010:cytoskeleton organization | GO:0030036:actin cytoskeleton organization |
| TDF307 | GO:0016020:membrane | GO:0005515:protein binding | GO:0016192:vesicle-mediated transport |  |
| TDF312 | GO:0000786:nucleosome | GO:0005634:nucleus | GO:0003677:DNA binding | GO:0006334:nucleosome assembly |
| TDF313 | GO:0016491:oxidoreductase activity | GO:0055114:oxidation reduction |  |  |
| TDF314 | GO:0004396:hexokinase activity | GO:0005524:ATP binding | GO:0016773:phosphotransferase activity, alcohol group as acceptor | GO:0005975:carbohydrate metabolic process |
|  | GO:0006096:glycolysis |  |  |  |
| TDF316 | GO:0005515:protein binding | GO:0005524:ATP binding | GO:0051082:unfolded protein binding | GO:0006457:protein folding |
|  | GO:0044267:cellular protein metabolic process |  |  |  |
| TDF318 | GO:0043022:ribosome binding | GO:0042256:mature ribosome assembly |  |  |
| TDF321 | GO:0005622:intracellular | GO:0005840:ribosome | GO:0003735:structural constituent of ribosome | GO:0006412:translation |
| TDF323 | GO:0000287:magnesium ion binding | GO:0003824:catalytic activity | GO:0004743:pyruvate kinase activity | GO:0030955:potassium ion binding |
|  | GO:0006096:glycolysis |  |  |  |
| TDF325 | GO:0003824:catalytic activity | GO:0005488:binding | GO:0016491:oxidoreductase activity | GO:0016615:malate dehydrogenase activity |
|  | GO:0016616:oxidoreductase activity, acting on the CH-OH group of donors, NAD or NADP as acceptor | GO:0030060:L-malate dehydrogenase activity | GO:0005975:carbohydrate metabolic process | GO:0006096:glycolysis |
|  | GO:0006108:malate metabolic process | GO:0008152:metabolic process | GO:0044262:cellular carbohydrate metabolic process | GO:0055114:oxidation reduction |
|  | GO:0008152:metabolic process | GO:0044262:cellular carbohydrate metabolic process | GO:0055114:oxidation reduction |  |
| TDF327 | GO:0016021:integral to membrane | GO:0006810:transport |  |  |
| TDF336 | GO:0005622:intracellular | GO:0005840:ribosome | GO:0003735:structural constituent of ribosome | GO:0006412:translation |
| TDF338 | GO:0045261:proton-transporting ATP synthase complex, catalytic core F(1) | GO:0046933:hydrogen ion transporting ATP synthase activity, rotational mechanism | GO:0046961:proton-transporting ATPase activity, rotational mechanism | GO:0015986:ATP synthesis coupled proton transport |
| TDF342 | GO:0016020:membrane | GO:0016021:integral to membrane | GO:0005215:transporter activity | GO:0005315:inorganic phosphate transmembrane transporter activity |
|  | GO:0006810:transport | GO:0006817:phosphate transport | GO:0055085:transmembrane transport |  |
| TDF355 | GO:0004497:monooxygenase activity | GO:0005506:iron ion binding | GO:0009055:electron carrier activity | GO:0020037:heme binding |
|  | GO:0055114:oxidation reduction |  |  |  |
| TDF364 | GO:0004497:monooxygenase activity | GO:0005506:iron ion binding | GO:0009055:electron carrier activity | GO:0020037:heme binding |
| TDF375 | GO:0016020:membrane | GO:0016021:integral to membrane | GO:0000287:magnesium ion binding | GO:0004012:phospholipid-translocating ATPase activity |
|  | GO:0005524:ATP binding | GO:0015662:ATPase activity, coupled to transmembrane movement of ions, phosphorylative mechanism | GO:0016820:hydrolase activity, acting on acid anhydrides, catalyzing transmembrane movement of substances | GO:0006754:ATP biosynthetic process |
|  | GO:0015914:phospholipid transport |  |  |  |
| TDF376 | GO:0016787:hydrolase activity | GO:0008152:metabolic process |  |  |
| TDF381 | GO:0003824:catalytic activity | GO:0004499:flavin-containing monooxygenase activity | GO:0005488:binding | GO:0050660:FAD binding |
|  | GO:0050661:NADP or NADPH binding | GO:0008152:metabolic process | GO:0055114:oxidation reduction |  |
| TDF383 | GO:0016020:membrane | GO:0016021:integral to membrane | GO:0008233:peptidase activity | GO:0008236:serine-type peptidase activity |
|  | GO:0006465:signal peptide processing | GO:0006508:proteolysis |  |  |
| TDF384 | GO:0005506:iron ion binding | GO:0051536:iron-sulfur cluster binding | GO:0016226:iron-sulfur cluster assembly |  |
| TDF385 | GO:0005622:intracellular | GO:0005840:ribosome | GO:0003735:structural constituent of ribosome | GO:0006412:translation |
