# Supplementary material for: Profiling and Quantifying Differential Gene Transcription Provide Insights into Ganoderic Acid Biosynthesis in Ganoderma lucidum in Response to Methyl Jasmonate
Source: PLoS One. 2013 Jun 7;8(6):e65027. doi: 10.1371/journal.pone.0065027 (PMC3676390; doi:10.1371/journal.pone.0065027)
Supplement: Table S2 — Pathway description of TDFs by searching the KEGG PATHWAY database. (DOC) [file pone.0065027.s005.doc]

Ang Ren, *et.al*., supplemental material file: Table S2

Table S2 Pathway description of TDFs by searching the KEGG PATHWAY database

|  | Seq. Name | Pathway | KO | Enzyme | Enzyme codes |  |
| --- | --- | --- | --- | --- | --- | --- |
| 1 | TDF006 | N-Glycan biosynthesis | K01228 | glucosidase I | EC:3.2.1.106 |  |
|  |  | Protein processing in endoplasmic reticulum |  |  |  |  |
|  |  | Metabolic pathways |  |  |  |  |
| 2 | TDF058 | Glycerophospholipid metabolism | K14676 | lysophospholipid hydrolase | EC:3.1.1.5 |  |
| 3 | TDF070 | Sphingolipid metabolism | K01441 | alkaline ceramidase | EC:3.5.1.23 |  |
|  |  | Metabolic pathways |  |  |  |  |
| 4 | TDF080 | Starch and sucrose metabolism | K00706 | 1,3-beta-glucan synthase | EC:2.4.1.34 |  |
| 5 | TDF113 | Metabolic pathways | K00626 | acetyl-CoA C-acetyltransferase | EC:2.3.1.9 |  |
|  |  | Fatty acid metabolism |  |  |  |  |
|  |  | Synthesis and degradation of ketone bodies |  |  |  |  |
|  |  | Valine, leucine and isoleucine degradation |  |  |  |  |
|  |  | Lysine degradation |  |  |  |  |
|  |  | Terpenoid backbone biosynthesis |  |  |  |  |
|  |  | Microbial metabolism in diverse environments |  |  |  |  |
|  |  | Biosynthesis of secondary metabolites |  |  |  |  |
| 6 | TDF115 | Biotin metabolism | K01942 | biotin--protein ligase | EC:6.3.4.9 |  |
|  |  | Metabolic pathways |  |  |  |  |
| 7 | TDF129 | Tryptophan metabolism | K03781 | catalase | EC:1.11.1.6 |  |
|  |  | Glyoxylate and dicarboxylate metabolism |  |  |  |  |
|  |  | Methane metabolism |  |  |  |  |
|  |  | Metabolic pathways |  |  |  |  |
|  |  | Biosynthesis of secondary metabolites |  |  |  |  |
|  |  | Microbial metabolism in diverse environments |  |  |  |  |
|  |  | Peroxisome |  |  |  |  |
| 8 | TDF138 | Phenylalanine, tyrosine and tryptophan biosynthesis | K13501 | anthranilate synthase | EC:4.1.3.27 |  |
|  |  | Metabolic pathways |  |  |  |  |
|  |  | Biosynthesis of secondary metabolites |  |  |  |  |
| 9 | TDF142 | Lysine biosynthesis | K00290 | saccharopine dehydrogenase (NAD+, L-lysine forming) | EC:1.5.1.7 |  |
|  |  | Lysine degradation |  |  |  |  |
|  |  | Metabolic pathways |  |  |  |  |
|  |  | Biosynthesis of secondary metabolites |  |  |  |  |
| 10 | TDF161 | Metabolic pathways | K01187 | alpha-glucosidase | EC:3.2.1.20 |  |
|  |  | Starch and sucrose metabolism |  |  |  |  |
|  |  | Galactose metabolism |  |  |  |  |
| 11 | TDF182 | Pyrimidine metabolism | K01520 | dUTP pyrophosphatase | EC:3.6.1.23 |  |
|  |  | Metabolic pathways |  |  |  |  |
| 12 | TDF195 | Citrate cycle (TCA cycle) | K01679 | fumarate hydratase | EC:4.2.1.2 |  |
|  |  | Metabolic pathways |  |  |  |  |
|  |  | Biosynthesis of secondary metabolites（12） |  |  |  |  |
|  |  | Microbial metabolism in diverse environments |  |  |  |  |
| 13 | TDF223 | Cyanoamino acid metabolism | K05349 | beta-glucosidase | EC:3.2.1.21 |  |
|  |  | Starch and sucrose metabolism |  |  |  |  |
|  |  | Metabolic pathways |  |  |  |  |
|  |  | Biosynthesis of secondary metabolites |  |  |  |  |
|  |  | Phenylpropanoid biosynthesis |  |  |  |  |
| 14 | TDF243 | Citrate cycle (TCA cycle) | K01958 | pyruvate carboxylase | EC:6.4.1.1 |  |
|  |  | Pyruvate metabolism |  |  |  |  |
|  |  | Metabolic pathways |  |  |  |  |
|  |  | Microbial metabolism in diverse environments |  |  |  |  |
| 15 | TDF291 | Microbial metabolism in diverse environments | K00121 | S-(hydroxymethyl)glutathione dehydrogenase | EC:1.1.1.284 |  |
|  |  | Metabolic pathways |  |  |  |  |
|  |  | Drug metabolism-cytochrome P450 |  |  |  |  |
|  |  | Chloroalkane and chloroalkene degradation |  |  |  |  |
|  |  | Naphthalene degradation |  |  |  |  |
|  |  | Glycolysis / Gluconeogenesis |  |  |  |  |
|  |  | Fatty acid metabolism |  |  |  |  |
|  |  | Tyrosine metabolism |  |  |  |  |
|  |  | Metabolism of xenobiotics by cytochrome P450 |  |  |  |  |
|  |  | Biosynthesis of secondary metabolites |  |  |  |  |
|  |  | Retinol metabolism |  |  |  |  |
| 16 | TDF300 | Metabolic pathways | K00927 | phosphoglycerate kinase | EC:2.7.2.3 |  |
|  |  | Glycolysis / Gluconeogenesis |  |  |  |  |
|  |  | Carbon fixation in photosynthetic organisms |  |  |  |  |
|  |  | Biosynthesis of secondary metabolites |  |  |  |  |
|  |  | Microbial metabolism in diverse environments |  |  |  |  |
| 17 | TDF313 | Amino sugar and nucleotide sugar metabolism | K00326 | cytochrome-b5 reductase | EC:1.6.2.2 |  |
|  |  | Biosynthesis of secondary metabolites |  |  |  |  |
| 18 | TDF314 | Amino sugar and nucleotide sugar metabolism | K00844 | hexokinase | EC:2.7.1.1 |  |
|  |  | Biosynthesis of secondary metabolites |  |  |  |  |
|  |  | Metabolic pathways |  |  |  |  |
|  |  | Glycolysis / Gluconeogenesis |  |  |  |  |
|  |  | Fructose and mannose metabolism |  |  |  |  |
|  |  | Starch and sucrose metabolism |  |  |  |  |
|  |  | Microbial metabolism in diverse environments |  |  |  |  |
|  |  | Galactose metabolism |  |  |  |  |
|  |  | Streptomycin biosynthesis |  |  |  |  |
|  |  | Butirosin and neomycin biosynthesis |  |  |  |  |
| 19 | TDF323 | Metabolic pathways | K00873 | pyruvate kinase | EC:2.7.1.40 |  |
|  |  | Purine metabolism |  |  |  |  |
|  |  | Biosynthesis of secondary metabolites |  |  |  |  |
|  |  | Microbial metabolism in diverse environments |  |  |  |  |
|  |  | Glycolysis / Gluconeogenesis |  |  |  |  |
|  |  | Pyruvate metabolism |  |  |  |  |
|  |  | Carbon fixation in photosynthetic organisms |  |  |  |  |
| 20 | TDF325 | Microbial metabolism in diverse environments | K00026 | malate dehydrogenase | EC:1.1.1.37 |  |
|  |  | Carbon fixation in photosynthetic organisms |  |  |  |  |
|  |  | Metabolic pathways |  |  |  |  |
|  |  | Citrate cycle (TCA cycle) |  |  |  |  |
|  |  | Biosynthesis of secondary metabolites |  |  |  |  |
| 21 | TDF338 | Oxidative phosphorylation | K02136 | F-type H+-transporting ATPase subunit gamma | EC:3.6.3.14 |  |
|  |  | Metabolic pathways |  |  |  |  |
