# Supplementary material for: Profiling and Quantifying Differential Gene Transcription Provide Insights into Ganoderic Acid Biosynthesis in Ganoderma lucidum in Response to Methyl Jasmonate
Source: PLoS One. 2013 Jun 7;8(6):e65027. doi: 10.1371/journal.pone.0065027 (PMC3676390; doi:10.1371/journal.pone.0065027)
Supplement: Table S3 — The genes expressed across MeJA-induction and the different developmental stages. (DOC) [file pone.0065027.s006.doc]

Ang Ren, *et.al*., supplemental material file: Table S3

Table S3 The genes expressed across MeJA-induction and the different developmental stages

| TDF | Mycelia EST library^a^ | Fruiting body EST library^b^ | A^c^ | B^d^ | C^e^ | D^f^ |
| --- | --- | --- | --- | --- | --- | --- |
| TDF001 | + | + |  |  |  | d |
| TDF002 | + | - |  | b |  |  |
| TDF003 | - | + |  |  | c |  |
| TDF004 | - | + |  |  | c |  |
| TDF005 | - | - | a |  |  |  |
| TDF006 | - | + |  |  | c |  |
| TDF007 | + | + |  |  |  | d |
| TDF008 | + | + |  |  |  | d |
| TDF009 | + | + |  |  |  | d |
| TDF010 | + | + |  |  |  | d |
| TDF011 | - | - | a |  |  |  |
| TDF012 | - | + |  |  | c |  |
| TDF013 | + | + |  |  |  | d |
| TDF014 | + | + |  |  |  | d |
| TDF015 | + | + |  |  |  | d |
| TDF016 | + | + |  |  |  | d |
| TDF017 | + | + |  |  |  | d |
| TDF018 | + | + |  |  |  | d |
| TDF019 | + | + |  |  |  | d |
| TDF020 | + | + |  |  |  | d |
| TDF021 | + | + |  |  |  | d |
| TDF022 | + | + |  |  |  | d |
| TDF023 | + | + |  |  |  | d |
| TDF024 | + | + |  |  |  | d |
| TDF025 | + | + |  |  |  | d |
| TDF026 | - | - | a |  |  |  |
| TDF027 | - | - | a |  |  |  |
| TDF028 | + | + |  |  |  | d |
| TDF029 | + | - |  | b |  |  |
| TDF030 | + | - |  | b |  |  |
| TDF031 | + | - |  | b |  |  |
| TDF032 | - | + |  |  | c |  |
| TDF033 | + | + |  |  |  | d |
| TDF034 | + | + |  |  |  | d |
| TDF035 | - | - | a |  |  |  |
| TDF036 | - | + |  |  | c |  |
| TDF037 | + | + |  |  |  | d |
| TDF038 | - | - | a |  |  |  |
| TDF039 | - | - | a |  |  |  |
| TDF040 | + | + |  |  |  | d |
| TDF041 | + | + |  |  |  | d |
| TDF042 | - | - | a |  |  |  |
| TDF043 | + | - |  | b |  |  |
| TDF044 | + | + |  |  |  | d |
| TDF045 | + | + |  |  |  | d |
| TDF046 | + | + |  |  |  | d |
| TDF047 | + | + |  |  |  | d |
| TDF048 | + | + |  |  |  | d |
| TDF049 | + | + |  |  |  | d |
| TDF050 | - | + |  |  | c |  |
| TDF051 | + | - |  | b |  |  |
| TDF052 | + | + |  |  |  | d |
| TDF053 | + | + |  |  |  | d |
| TDF054 | + | + |  |  |  | d |
| TDF055 | + | + |  |  |  | d |
| TDF056 | + | + |  |  |  | d |
| TDF057 | + | + |  |  |  | d |
| TDF058 | + | + |  |  |  | d |
| TDF059 | + | + |  |  |  | d |
| TDF060 | + | + |  |  |  | d |
| TDF061 | + | + |  |  |  | d |
| TDF062 | - | - | a |  |  |  |
| TDF063 | + | + |  |  |  | d |
| TDF064 | - | + |  |  | c |  |
| TDF065 | - | + |  |  | c |  |
| TDF066 | - | + |  |  | c |  |
| TDF067 | - | + |  |  | c |  |
| TDF068 | - | + |  |  | c |  |
| TDF069 | - | - | a |  |  |  |
| TDF070 | + | + |  |  |  | d |
| TDF071 | + | + |  |  |  | d |
| TDF072 | + | + |  |  |  | d |
| TDF073 | + | + |  |  |  | d |
| TDF074 | - | - | a |  |  |  |
| TDF075 | - | - | a |  |  |  |
| TDF076 | - | + |  |  | c |  |
| TDF077 | - | - | a |  |  |  |
| TDF078 | + | + |  |  |  | d |
| TDF079 | + | + |  |  |  | d |
| TDF080 | + | + |  |  |  | d |
| TDF081 | + | + |  |  |  | d |
| TDF082 | + | + |  |  |  | d |
| TDF083 | + | + |  |  |  | d |
| TDF084 | + | + |  |  |  | d |
| TDF085 | + | + |  |  |  | d |
| TDF086 | - | - | a |  |  |  |
| TDF087 | + | + |  |  |  | d |
| TDF088 | - | - | a |  |  |  |
| TDF089 | - | - | a |  |  |  |
| TDF090 | - | + |  |  | c |  |
| TDF091 | - | - | a |  |  |  |
| TDF092 | + | + |  |  |  | d |
| TDF093 | + | + |  |  |  | d |
| TDF094 | + | + |  |  |  | d |
| TDF095 | + | + |  |  |  | d |
| TDF096 | + | + |  |  |  | d |
| TDF097 | - | - | a |  |  |  |
| TDF098 | + | + |  |  |  | d |
| TDF099 | - | + |  |  | c |  |
| TDF100 | - | + |  |  | c |  |
| TDF101 | + | + |  |  |  | d |
| TDF102 | + | - |  | b |  |  |
| TDF103 | - | + |  |  | c |  |
| TDF104 | + | + |  |  |  | d |
| TDF105 | + | + |  |  |  | d |
| TDF106 | + | + |  |  |  | d |
| TDF107 | + | + |  |  |  | d |
| TDF108 | + | + |  |  |  | d |
| TDF109 | + | + |  |  |  | d |
| TDF110 | + | + |  |  |  | d |
| TDF111 | + | + |  |  |  | d |
| TDF112 | - | - | a |  |  |  |
| TDF113 | + | + |  |  |  | d |
| TDF114 | + | + |  |  |  | d |
| TDF115 | + | + |  |  |  | d |
| TDF116 | - | + |  |  | c |  |
| TDF117 | - | - | a |  |  |  |
| TDF118 | - | - | a |  |  |  |
| TDF119 | + | + |  |  |  | d |
| TDF120 | + | + |  |  |  | d |
| TDF121 | + | + |  |  |  | d |
| TDF122 | + | + |  |  |  | d |
| TDF123 | + | + |  |  |  | d |
| TDF124 | + | + |  |  |  | d |
| TDF125 | + | + |  |  |  | d |
| TDF126 | - | - | a |  |  |  |
| TDF127 | + | + |  |  |  | d |
| TDF128 | + | + |  |  |  | d |
| TDF129 | - | - | a |  |  |  |
| TDF130 | + | + |  |  |  | d |
| TDF131 | - | - | a |  |  |  |
| TDF132 | - | + |  |  | c |  |
| TDF133 | - | - | a |  |  |  |
| TDF134 | + | + |  |  |  | d |
| TDF135 | + | + |  |  |  | d |
| TDF136 | + | + |  |  |  | d |
| TDF137 | - | - | a |  |  |  |
| TDF138 | + | + |  |  |  | d |
| TDF139 | + | + |  |  |  | d |
| TDF140 | + | + |  |  |  | d |
| TDF141 | + | + |  |  |  | d |
| TDF142 | + | + |  |  |  | d |
| TDF143 | + | + |  |  |  | d |
| TDF144 | - | - | a |  |  |  |
| TDF145 | + | + |  |  |  | d |
| TDF146 | + | + |  |  |  | d |
| TDF147 | + | + |  |  |  | d |
| TDF148 | + | - |  | b |  |  |
| TDF149 | + | + |  |  |  | d |
| TDF150 | + | + |  |  |  | d |
| TDF151 | + | + |  |  |  | d |
| TDF152 | + | + |  |  |  | d |
| TDF153 | + | + |  |  |  | d |
| TDF154 | + | + |  |  |  | d |
| TDF155 | + | + |  |  |  | d |
| TDF156 | + | + |  |  |  | d |
| TDF157 | - | - | a |  |  |  |
| TDF158 | + | + |  |  |  | d |
| TDF159 | + | + |  |  |  | d |
| TDF160 | - | + |  |  | c |  |
| TDF161 | - | + |  |  | c |  |
| TDF162 | + | + |  |  |  | d |
| TDF163 | - | + |  |  | c |  |
| TDF164 | + | + |  |  |  | d |
| TDF165 | + | + |  |  |  | d |
| TDF166 | - | + |  |  | c |  |
| TDF167 | - | + |  |  | c |  |
| TDF168 | + | + |  |  |  | d |
| TDF169 | + | + |  |  |  | d |
| TDF170 | + | + |  |  |  | d |
| TDF171 | + | + |  |  |  | d |
| TDF172 | - | + |  |  | c |  |
| TDF173 | + | + |  |  |  | d |
| TDF174 | - | + |  |  | c |  |
| TDF175 | + | + |  |  |  | d |
| TDF176 | + | + |  |  |  | d |
| TDF177 | + | + |  |  |  | d |
| TDF178 | + | + |  |  |  | d |
| TDF179 | - | - | a |  |  |  |
| TDF180 | + | + |  |  |  | d |
| TDF181 | + | + |  |  |  | d |
| TDF182 | + | + |  |  |  | d |
| TDF183 | - | - | a |  |  |  |
| TDF184 | + | + |  |  |  | d |
| TDF185 | + | - |  | b |  |  |
| TDF186 | - | - | a |  |  |  |
| TDF187 | + | + |  |  |  | d |
| TDF188 | + | + |  |  |  | d |
| TDF189 | + | + |  |  |  | d |
| TDF190 | + | + |  |  |  | d |
| TDF191 | - | + |  |  | c |  |
| TDF192 | - | - | a |  |  |  |
| TDF193 | + | + |  |  |  | d |
| TDF194 | + | + |  |  |  | d |
| TDF195 | + | + |  |  |  | d |
| TDF196 | - | - | a |  |  |  |
| TDF197 | + | + |  |  |  | d |
| TDF198 | + | + |  |  |  | d |
| TDF199 | - | + |  |  | c |  |
| TDF200 | + | + |  |  |  | d |
| TDF201 | + | + |  |  |  | d |
| TDF202 | + | + |  |  |  | d |
| TDF203 | + | + |  |  |  | d |
| TDF204 | + | + |  |  |  | d |
| TDF205 | + | - |  | b |  |  |
| TDF206 | - | - | a |  |  |  |
| TDF207 | + | - |  | b |  |  |
| TDF208 | + | - |  | b |  |  |
| TDF209 | - | + |  |  | c |  |
| TDF210 | - | + |  |  | c |  |
| TDF211 | + | + |  |  |  | d |
| TDF212 | + | + |  |  |  | d |
| TDF213 | + | + |  |  |  | d |
| TDF214 | - | + |  |  | c |  |
| TDF215 | + | + |  |  |  | d |
| TDF216 | - | - | a |  |  |  |
| TDF217 | + | + |  |  |  | d |
| TDF218 | + | - |  | b |  |  |
| TDF219 | + | + |  |  |  | d |
| TDF220 | - | + |  |  | c |  |
| TDF221 | - | + |  |  | c |  |
| TDF222 | + | + |  |  |  | d |
| TDF223 | - | + |  |  | c |  |
| TDF224 | + | + |  |  |  | d |
| TDF225 | + | + |  |  |  | d |
| TDF226 | + | + |  |  |  | d |
| TDF227 | + | + |  |  |  | d |
| TDF228 | - | + |  |  | c |  |
| TDF229 | + | - |  | b |  |  |
| TDF230 | + | + |  |  |  | d |
| TDF231 | + | + |  |  |  | d |
| TDF232 | + | + |  |  |  | d |
| TDF233 | + | + |  |  |  | d |
| TDF234 | - | - | a |  |  |  |
| TDF235 | + | + |  |  |  | d |
| TDF236 | + | - |  | b |  |  |
| TDF237 | - | - | a |  |  |  |
| TDF238 | - | + |  |  | c |  |
| TDF239 | + | + |  |  |  | d |
| TDF240 | + | + |  |  |  | d |
| TDF241 | + | - |  | b |  |  |
| TDF242 | - | - | a |  |  |  |
| TDF243 | + | + |  |  |  | d |
| TDF244 | + | + |  |  |  | d |
| TDF245 | + | + |  |  |  | d |
| TDF246 | - | - | a |  |  |  |
| TDF247 | - | - | a |  |  |  |
| TDF248 | + | + |  |  |  | d |
| TDF249 | + | + |  |  |  | d |
| TDF250 | + | - |  | b |  |  |
| TDF251 | + | + |  |  |  | d |
| TDF252 | - | - | a |  |  |  |
| TDF253 | + | + |  |  |  | d |
| TDF254 | + | + |  |  |  | d |
| TDF255 | + | + |  |  |  | d |
| TDF256 | - | + |  |  | c |  |
| TDF257 | + | + |  |  |  | d |
| TDF258 | + | - |  | b |  |  |
| TDF259 | + | + |  |  |  | d |
| TDF260 | + | + |  |  |  | d |
| TDF261 | - | + |  |  | c |  |
| TDF262 | - | - | a |  |  |  |
| TDF263 | + | - |  | b |  |  |
| TDF264 | + | + |  |  |  | d |
| TDF265 | + | + |  |  |  | d |
| TDF266 | - | - | a |  |  |  |
| TDF267 | + | + |  |  |  | d |
| TDF268 | - | - | a |  |  |  |
| TDF269 | + | + |  |  |  | d |
| TDF270 | + | - |  | b |  |  |
| TDF271 | - | - | a |  |  |  |
| TDF272 | + | - |  | b |  |  |
| TDF273 | - | - | a |  |  |  |
| TDF274 | + | - |  | b |  |  |
| TDF275 | - | + |  |  | c |  |
| TDF276 | + | + |  |  |  | d |
| TDF277 | + | + |  |  |  | d |
| TDF278 | + | + |  |  |  | d |
| TDF279 | - | - | a |  |  |  |
| TDF280 | + | - |  | b |  |  |
| TDF281 | + | - |  | b |  |  |
| TDF282 | + | + |  |  |  | d |
| TDF283 | - | - | a |  |  |  |
| TDF284 | + | + |  |  |  | d |
| TDF285 | - | - | a |  |  |  |
| TDF286 | + | + |  |  |  | d |
| TDF287 | - | - | a |  |  |  |
| TDF288 | + | + |  |  |  | d |
| TDF289 | + | + |  |  |  | d |
| TDF290 | + | - |  | b |  |  |
| TDF291 | + | + |  |  |  | d |
| TDF292 | + | + |  |  |  | d |
| TDF293 | + | + |  |  |  | d |
| TDF294 | + | + |  |  |  | d |
| TDF295 | + | - |  | b |  |  |
| TDF296 | + | - |  | b |  |  |
| TDF297 | + | + |  |  |  | d |
| TDF298 | + | + |  |  |  | d |
| TDF299 | + | + |  |  |  | d |
| TDF300 | + | + |  |  |  | d |
| TDF301 | + | + |  |  |  | d |
| TDF302 | + | + |  |  |  | d |
| TDF303 | + | + |  |  |  | d |
| TDF304 | + | + |  |  |  | d |
| TDF305 | + | + |  |  |  | d |
| TDF306 | + | + |  |  |  | d |
| TDF307 | + | + |  |  |  | d |
| TDF308 | + | + |  |  |  | d |
| TDF309 | + | + |  |  |  | d |
| TDF310 | + | + |  |  |  | d |
| TDF311 | - | + |  |  | c |  |
| TDF312 | + | + |  |  |  | d |
| TDF313 | + | + |  |  |  | d |
| TDF314 | + | + |  |  |  | d |
| TDF315 | + | + |  |  |  | d |
| TDF316 | + | + |  |  |  | d |
| TDF317 | + | + |  |  |  | d |
| TDF318 | + | + |  |  |  | d |
| TDF319 | - | + |  |  | c |  |
| TDF320 | + | + |  |  |  | d |
| TDF321 | + | + |  |  |  | d |
| TDF322 | + | + |  |  |  | d |
| TDF323 | - | + |  |  | c |  |
| TDF324 | + | + |  |  |  | d |
| TDF325 | + | + |  |  |  | d |
| TDF326 | + | + |  |  |  | d |
| TDF327 | + | + |  |  |  | d |
| TDF328 | + | + |  |  |  | d |
| TDF329 | + | + |  |  |  | d |
| TDF330 | + | + |  |  |  | d |
| TDF331 | + | + |  |  |  | d |
| TDF332 | + | + |  |  |  | d |
| TDF333 | + | + |  |  |  | d |
| TDF334 | + | + |  |  |  | d |
| TDF335 | + | + |  |  |  | d |
| TDF336 | + | + |  |  |  | d |
| TDF337 | + | + |  |  |  | d |
| TDF338 | + | + |  |  |  | d |
| TDF339 | + | - |  | b |  |  |
| TDF340 | + | + |  |  |  | d |
| TDF341 | + | + |  |  |  | d |
| TDF342 | + | + |  |  |  | d |
| TDF343 | + | + |  |  |  | d |
| TDF344 | + | + |  |  |  | d |
| TDF345 | + | + |  |  |  | d |
| TDF346 | + | + |  |  |  | d |
| TDF347 | + | + |  |  |  | d |
| TDF348 | + | - |  | b |  |  |
| TDF349 | + | + |  |  |  | d |
| TDF350 | + | + |  |  |  | d |
| TDF351 | + | + |  |  |  | d |
| TDF352 | - | - | a |  |  |  |
| TDF353 | + | + |  |  |  | d |
| TDF354 | + | + |  |  |  | d |
| TDF355 | - | + |  |  | c |  |
| TDF356 | - | + |  |  | c |  |
| TDF357 | + | + |  |  |  | d |
| TDF358 | + | + |  |  |  | d |
| TDF359 | + | + |  |  |  | d |
| TDF360 | + | + |  |  |  | d |
| TDF361 | + | + |  |  |  | d |
| TDF362 | + | + |  |  |  | d |
| TDF363 | + | + |  |  |  | d |
| TDF364 | - | + |  |  | c |  |
| TDF365 | + | + |  |  |  | d |
| TDF366 | + | + |  |  |  | d |
| TDF367 | + | + |  |  |  | d |
| TDF368 | + | + |  |  |  | d |
| TDF369 | + | + |  |  |  | d |
| TDF370 | + | + |  |  |  | d |
| TDF371 | + | + |  |  |  | d |
| TDF372 | + | + |  |  |  | d |
| TDF373 | + | + |  |  |  | d |
| TDF374 | + | + |  |  |  | d |
| TDF375 | + | + |  |  |  | d |
| TDF376 | + | + |  |  |  | d |
| TDF377 | + | + |  |  |  | d |
| TDF378 | - | + |  |  | c |  |
| TDF379 | + | + |  |  |  | d |
| TDF380 | - | + |  |  | c |  |
| TDF381 | - | + |  |  | c |  |
| TDF382 | + | + |  |  |  | d |
| TDF383 | + | + |  |  |  | d |
| TDF384 | + | + |  |  |  | d |
| TDF385 | + | - |  | b |  |  |
| TDF386 | - | + |  |  | c |  |
| TDF387 | + | + |  |  |  | d |
| TDF388 | + | + |  |  |  | d |
| TDF389 | + | + |  |  |  | d |
| TDF390 | + | + |  |  |  | d |

^a, b^ the mycelia EST libraryt from Chen et al., 2012, ^c^ the genes only appeared in the MeJA-induced EST library, ^d^ the genes appeared in both MeJA-induced EST library and mycelia EST library, ^e^ the genes appeared in both MeJA-induced EST library and fruiting body EST library, ^f^ the genes appeared in three EST libraries (MeJA-induced EST library, mycelia EST library and fruiting body EST library).
