# Supplementary material for: Profiling and Quantifying Differential Gene Transcription Provide Insights into Ganoderic Acid Biosynthesis in Ganoderma lucidum in Response to Methyl Jasmonate
Source: PLoS One. 2013 Jun 7;8(6):e65027. doi: 10.1371/journal.pone.0065027 (PMC3676390; doi:10.1371/journal.pone.0065027)
Supplement: Table S4 — Primer sets used for pre-amplified and selective amplified primers. (DOC) [file pone.0065027.s007.doc]

Ang Ren, *et.al*., supplemental material file: Table S4

Table S4 Primer sets used for pre-amplified and selective amplified primers

| Primer | Sequence (5’-3’) |
| --- | --- |
| EcoR-00 | GTAGACTGCGTACCAATTC |
| MseI-00 | GATGAGTCCTGAGTAA |
| EcoR-13 | GTAGACTGCGTACCAATTCAC |
| EcoR-14 | GTAGACTGCGTACCAATTCAG |
| EcoR-23 | GTAGACTGCGTACCAATTCTC |
| EcoR-24 | GTAGACTGCGTACCAATTCTG |
| EcoR-33 | GTAGACTGCGTACCAATTCCC |
| EcoR-41 | GTAGACTGCGTACCAATTCGA |
| EcoR-42 | GTAGACTGCGTACCAATTCGT |
| EcoR-44 | GTAGACTGCGTACCAATTCGG |
| MseI-12 | GATGAGTCCTGAGTAAAT |
| MseI-23 | GATGAGTCCTGAGTAATC |
| MseI-24 | GATGAGTCCTGAGTAATG |
| MseI-32 | GATGAGTCCTGAGTAACT |
| MseI-33 | GATGAGTCCTGAGTAACC |
| MseI-34 | GATGAGTCCTGAGTAAAG |
| MseI-43 | GATGAGTCCTGAGTAAAC |
| MseI-44 | GATGAGTCCTGAGTAAGG |
