# Supplementary material for: Profiling and Quantifying Differential Gene Transcription Provide Insights into Ganoderic Acid Biosynthesis in Ganoderma lucidum in Response to Methyl Jasmonate
Source: PLoS One. 2013 Jun 7;8(6):e65027. doi: 10.1371/journal.pone.0065027 (PMC3676390; doi:10.1371/journal.pone.0065027)
Supplement: Table S5 — Primer sets used for quantitative real-time PCR. (DOC) [file pone.0065027.s008.doc]

Ang Ren, *et.al*., supplemental material file: Table S5

Table S5 Primer sets used for quantitative real-time PCR

| Target gene | Primer | Sequence | Predicted product size(bp) |
| --- | --- | --- | --- |
| *cmpk* (TDF009) | Forward | 5′-CTTTCTTGCCGACGGATTT-3′ | 189 |
|  | Reverse | 5′-CCACCCTTACCTTGCTGTTT-3′ |  |
| *nbp* (TDF013) | Forward | 5′-CGGAAGAACCTGAACTAACCC-3′ | 145 |
|  | Reverse | 5′-ATGAGCCCTCGCCATACAA-3′ |  |
| *ndd* (TDF015) | Forward | 5′-GAGCGATGACGGACGAGTA-3′ | 140 |
|  | Reverse | 5′-GGAATCAACAGCCAGAGGG-3′ |  |
| *apk* (TDF040) | Forward | 5′-CACGCCGTCGTCATCTTTC-3′ | 100 |
|  | Reverse | 5′-CACCTCGTGCTTCCAGACC-3′ |  |
| *prp* (TDF042) | Forward | 5′-ATGAAGTCCGCCGTGTTCG-3′ | 132 |
|  | Reverse | 5′-CTGCTGCTGATGGATCTGTCC-3′ |  |
| *cyt* (TDF047) | Forward | 5′-GCGAAGGCGGTTGGTTTAG-3′ | 178 |
|  | Reverse | 5′-GCTGCCAGTTGACTTGCTC-3′ |  |
| *hk* (TDF051) | Forward | 5′-CCACTCCAGAGGTGGTGCT-3′ | 194 |
|  | Reverse | 5′-ATGACGACATCCCAGACGAA-3′ |  |
| *mob* (TDF052) | Forward | 5′-GGGAGCATCACAATCGTCTT-3′ | 164 |
|  | Reverse | 5′-CCCACCTCTGCCAACTTTC-3′ |  |
| *nuc* (TDF058) | Forward | 5′-GTATCCTCCATCCACAAGCA-3′ | 132 |
|  | Reverse | 5′-CCGAGTAATCCCTCCCAAAT-3′ |  |
| *guf* (TDF078) | Forward | 5′-ACCTGGGAGCGAGTTAGGC-3′ | 166 |
|  | Reverse | 5′-ATCCGATTTGGAAGATAGACGA-3′ |  |
| *gls* (TDF080) | Forward | 5′-GGGCAAGTCGCGGAAGTAT-3′ | 107 |
|  | Reverse | 5′-CGAAGACGAGGAACCAGAGC-3′ |  |
| *aao* (TDF096) | Forward | 5′-GAGCAGCGTTGTTGACCCC-3′ | 123 |
|  | Reverse | 5′-TCGGCAAATGCGTACACCA-3′ |  |
| *aat* (TDF113) | Forward | 5′-TGGGGTTGAGGGTAGGGAC-3′ | 125 |
|  | Reverse | 5′-GGCGGGTCAGTTCAAGGAG-3′ |  |
| *cdc* (TDF122) | Forward | 5′-CCGAATACAAGGAAGGGAA-3′ | 190 |
|  | Reverse | 5′-AGTCTGTGGCGACAAGCAA-3′ |  |
| *rho* (TDF165) | Forward | 5′-TTCGGTTAGATGGCAAGGC-3′ | 141 |
|  | Reverse | 5′-CAAGAGAATCGGGCGTGTC-3′ |  |
| *pco* (TDF243) | Forward | 5′-AAGGTCGTCGGAGATTTCG-3′ | 161 |
|  | Reverse | 5′-CTACGGAGAGGCTCTGGGA-3′ |  |
| *ksr* (TDF256) | Forward | 5′-CAGCACGCTCTTGACCGAC-3′ | 130 |
|  | Reverse | 5′-CAACCTCCTGGACGAACAC-3′ |  |
| *vmp* (TDF264) | Forward | 5′-CACCAGGTTCCCGTGTTAT-3′ | 173 |
|  | Reverse | 5′-TTGCCAGTCCCTCCTTTAC-3′ |  |
| *gfd* (TDF291) | Forward | 5′-TCCACTGTCGCTGTCTTCG-3′ | 122 |
|  | Reverse | 5′-CGTGCCCATGACTCCTTTC-3′ |  |
| *hd* (TDF293) | Forward | 5′-CATAAACTGGGCAGGCGGTCT-3′ | 110 |
|  | Reverse | 5′-GGGGAACGTAGCGAAGCAT-3′ |  |
| *cal* (TDF375) | Forward | 5′-ATCAAGAGTCAACGCAGCAC-3′ | 172 |
|  | Reverse | 5′-AGATCGCAAGCTCTAAAAA-3′ |  |
| *cat* (TDF129) | Forward | 5′-GGGAGGGTCAACGCAGCAC-3′ | 112 |
|  | Reverse | 5′-AACGCCTCTCGCACCCAGT-3′ |  |
| *fum* (TDF195) | Forward | 5′-GGCGACCATCCTTAACAGC-3′ | 167 |
|  | Reverse | 5′-CGTCGGGGTAAAGCATCAG-3′ |  |
| *pyr* (TDF323) | Forward | 5′-TGTTGCGAATGCCGTGATG-3′ | 180 |
|  | Reverse | 5′-TCCGTGGGACGAGAAGTGG-3′ |  |
| *vac* (TDF360) | Forward | 5′-CCACCTGGTCCGCATCAAC-3′ | 184 |
|  | Reverse | 5′-CGCTTCTTCGCAGACATCG-3′ |  |
